# Chiral-at-metal catalysts: history, terminology, design, synthesis, and applications

**DOI:** 10.1039/d4cs01043d

**Published:** 2025-01-21

**Authors:** Lilu Zhang, Eric Meggers

**Affiliations:** a Fachbereich Chemie, Philipps-Universität Marburg, Hans-Meerwein-Strasse 4 35043 Marburg Germany meggers@chemie.uni-marburg.de

## Abstract

For decades, advances in chiral transition metal catalysis have been closely tied to the development of customized chiral ligands. Recently, however, an alternative approach to this traditional metal-plus-chiral-ligand method has emerged. In this new strategy, chiral transition metal catalysts are composed entirely of achiral ligands, with the overall chirality originating exclusively from a stereogenic metal center. This “chiral-at-metal” approach offers the benefit of structural simplicity. More importantly, by removing the need for chiral elements within the ligand framework, it opens up new possibilities for designing innovative catalyst architectures with unique properties. As a result, chiral-at-metal catalysis is becoming an increasingly important area of research. This review offers a comprehensive overview and detailed insights into asymmetric chiral-at-metal catalysis, encouraging scientists to explore new avenues in asymmetric transition metal catalysis and driving innovation in both fundamental and applied research.

## Abstract 2

Chiral-at-metal catalysts, containing only achiral ligands, utilize the metal as the sole stereogenic center and the catalytic center. They represent an emerging and powerful class of chiral transition metal catalysts for asymmetric catalysis.

## Graphical abstract

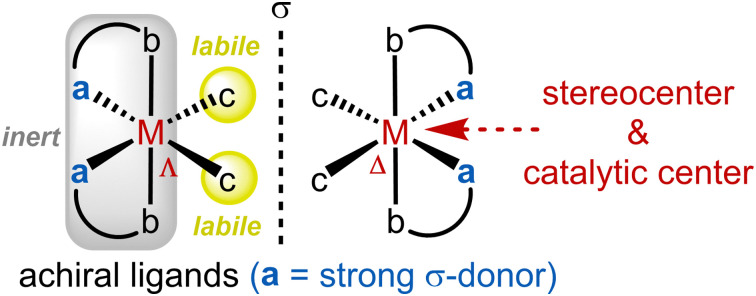

## 1. Introduction

### 1.1. Asymmetric catalysis

Asymmetric catalysis holds great promise as a highly cost-effective approach for producing enantiomerically pure chiral compounds, as a single chiral catalyst can amplify chiral information to produce many optically active chiral molecules.^1,2^ Chiral catalysts generally fall into three categories: biocatalysts (enzymes), chiral organic compounds (organocatalysts), and chiral metal complexes. It is unsurprising that chemists initially focused on developing synthetic chiral catalysts using transition metal complexes. The wide range of metals with varying oxidation states, diverse coordination geometries, and numerous ligand types allow transition metal catalysis to offer an extensive array of modes of activation and catalytic mechanisms.

### 1.2. Chiral ligands for chiral transition metal catalysts

**Fig. 1:**
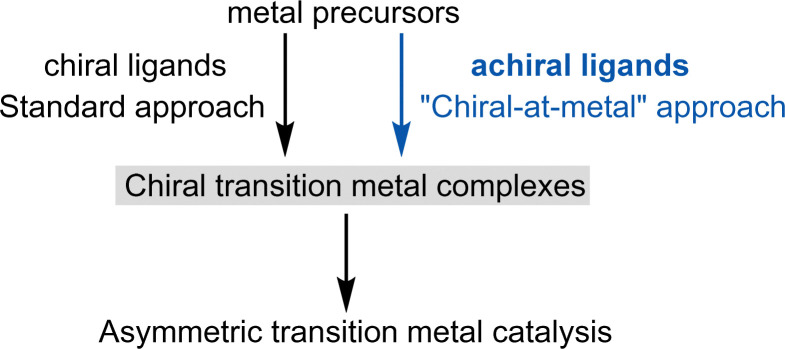
Chiral transition metal catalysis: chiral ligands *versus* achiral ligands for the design of chiral transition metal catalysts.

Asymmetric induction can be achieved when the metal complex itself is chiral. Traditionally, this chirality has been generated by combining metal salts or metal precursor complexes with one or more chiral ligands. This area of research, pioneered by Knowles,^3^ Noyori,^4^ Sharpless,^5^ Kagan,^6^ and others,^2^ has led to the development of numerous carefully tailored chiral ligands, including DuPhos, BINAP, PHOX, TADDOL, Salen, BOX, PyBOX, and Josiphos, among many others. These ligands, often referred to as “privileged chiral ligands,” have demonstrated exceptional versatility.^7^ As a result, chiral ligand design has been highly successful and central to asymmetric transition metal catalysis for over half a century (Fig. 1, left).

### 1.3. Achiral ligands for chiral transition metal catalysts

However, since Alfred Werner's seminal work over a century ago,^8^ which first characterized chiral metal complexes and introduced the concept of metal-centered chirality, it has been well-established that chiral metal complexes can be formed entirely from achiral ligands. Despite this knowledge, chiral-at-metal complexes were largely overlooked in asymmetric transition metal catalysis until our group revealed their broad utility (Fig. 1, right).

### 1.4. Terminology: “chirality-at-metal”

It is important to recognize that chiral metal complexes with chiral ligands often also feature a stereogenic metal center.^9^ To differentiate between chiral metal complexes that contain only achiral ligands and those that involve chiral ligands, we prefer the term “chiral-at-metal” (or more specifically, “chiral-at-iron”, “chiral-at-rhodium”, *etc.*). This terminology emphasizes that the overall chirality of the metal complex formally arises solely from the stereogenic metal center. Although the term “stereogenic-at-metal” has been used, we find it less precise, as a stereogenic metal center alone does not fully define the overall chirality.

## 2. Early work

### 2.1. Seminal work of Alfred Werner (1911)

**Fig. 2:**
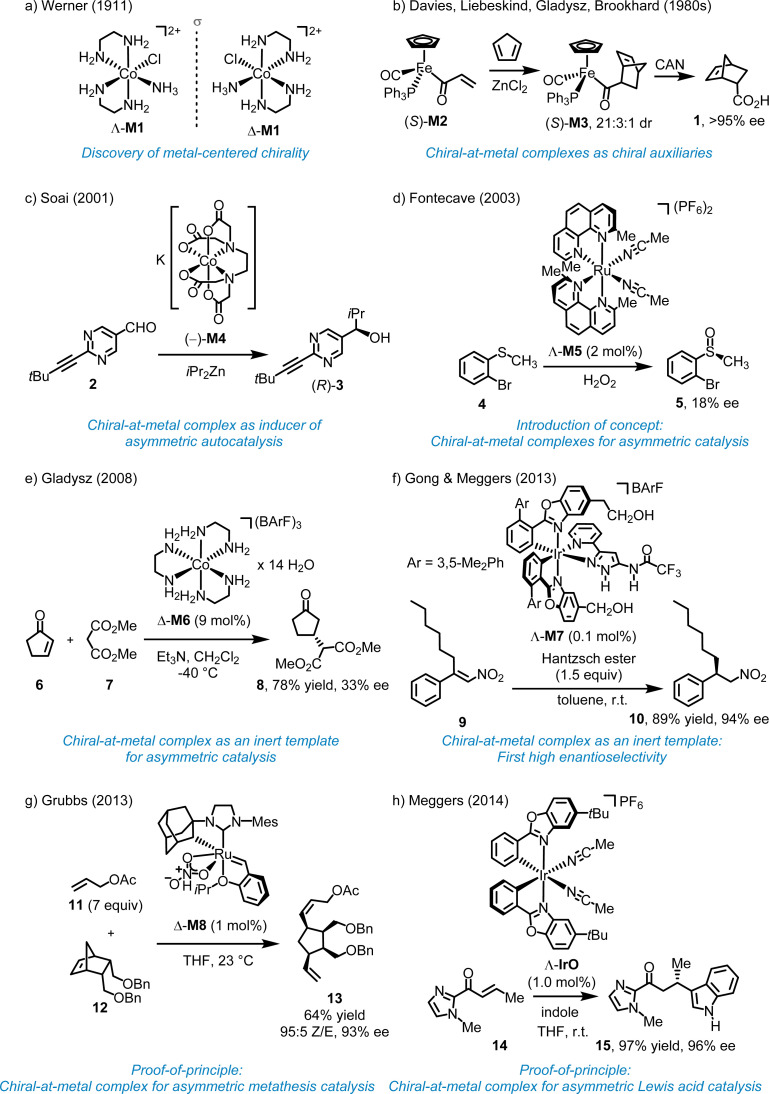
Early work on chiral-at-metal complexes and their applications.

More than 100 years ago, Werner demonstrated that the octahedral coordination complex *cis*-[Co(en)_2_(NH_3_)Cl]Cl_2_ (en = 1,2-ethylenediamine) (M1) is chiral despite containing solely achiral ligands (Fig. 2a).^10^ Various terms have been used to describe this source of chirality, including “metal-centered chirality,” “metal centrochirality,” “chiral metal,” “chiral-at-metal,” and “stereogenic-at-metal”. However, it is important to note that chirality formally refers to the geometrical properties of an entire compound rather than a specific structural element. In Werner's complexes, chirality is a result of the helical arrangement of the two 1,2-ethylenediamine ligands, which form either a left-handed (Λ-configuration at the metal) or right-handed (Δ-configuration at the metal) helix.

### 2.2. Chiral-at-metal complexes as chiral auxiliaries (1980s)

In 1969, Brunner reported the first optically active half-sandwich complex, featuring a central Mn ion asymmetrically coordinated to four distinct substituents in a pseudotetrahedral arrangement with sufficient configurational stability.^11^ In the 1980s, Davies, Gladysz, Brookhart, and Liebeskind initiated the first studies on related chiral-at-metal piano-stool half-sandwich complexes as tools for asymmetric organic synthesis, specifically using such chiral-at-metal complexes as chiral auxiliaries.^12–17^ For example, Davies demonstrated that the chiral-at-metal α,β-unsaturated acyl iron complex (*S*)-M2 can serve as a dienophile in the diastereoselective Diels–Alder reaction with cyclopentadiene, yielding the cycloaddition product (*S*)-M3.^12^ Following oxidative cleavage of the Fe–C bond using (NH_4_)_2_Ce(NO_3_)_6_ (CAN), *endo*-5-norbornene-2-carboxylic acid (1) was obtained with >95% enantiomeric excess (ee) (Fig. 2b). In this case, the enantiopure chiral-at-iron half-sandwich unit functioned as a chiral auxiliary for a diastereoselective cycloaddition.

### 2.3. Chiral-at-metal complexes as inducer for asymmetric autocatalysis (2001)

Soai and coworkers reported that chiral-at-cobalt complexes, such as the EDTA complex (−)-M4, in which the chirality arises solely from the octahedral metal stereocenter, can act as chiral inducer in the enantioselective addition of diisopropylzinc to pyrimidine-5-carbaldehyde 2, producing the chiral alcohol (*R*)-3 with up to 94% ee (Fig. 2c).^18^ This reaction follows an autocatalytic mechanism, with the chiral cobalt complex functioning as an inducer rather than as a catalyst.

### 2.4. First example of chiral-at-metal complex for asymmetric transition metal catalysis (2003)

In 2003, Fontecave proposed utilizing a reactive chiral-at-metal complex for asymmetric catalysis, with the metal acting simultaneously as the sole stereogenic center and the reactive site. Fontecave reported that *cis*-[Ru(2,9-Me_2_phen)_2_(MeCN)_2_] (PF_6_)_2_ (M5) catalyzes the oxidation of organic sulfides to sulfoxides (Fig. 2d).^19^ Although the ligands themselves are achiral, the *cis*-coordinated phenanthroline ligands induce helical chirality at the ruthenium center, resulting in either a Λ- or Δ-configured metal (left- or right-handed helix). However, the degree of asymmetric induction was very low, with the best result, the conversion 4 → 5, yielding only 18% ee, possibly due to a degradation of the ruthenium complex under the reaction conditions. As a result, while Fontecave introduced the concept of chiral-at-metal catalysis, the experiments did not serve as definitive proof-of-concept.

### 2.5. Chiral-at-metal complexes as inert templates for asymmetric catalysis (since 2008)

In 2008, Gladysz reported that the Δ-enantiomer of the simple cobalt(iii) complex [Co(en)_3_](BArF)_3_ (M6) with the large counterion tetrakis[3,5-bis(trifluoromethyl)phenyl]borate (BArF) catalyzes the Michael reaction of 2-cyclopenten-1-one (6) with dimethyl malonate (7) to provide the Michael adduct 8, although the enantioselectivity was low (33% ee) (Fig. 2e).^20^ Because of the inertness of the cobalt complex, catalysis must occur through interactions with the ligand sphere. In 2013, Gong and Meggers demonstrated the merit of this approach by introducing the chiral-at-metal complex Λ-M7 as a highly efficient catalyst for the enantioselective hydrogenation of nitro alkenes using Hantzsch ester (Fig. 2f).^21^ For instance, the β,β-disubstituted nitroalkene 9 was reduced to the corresponding nitroalkane 10 with 94% ee and 89% yield, using just 0.1 mol% of the chiral-at-iridium catalyst Λ-M7. Mechanistically, since the iridium complex is completely substitutionally inert, the metal center functions solely as a structural center, with catalysis occurring through the ligand sphere *via* hydrogen bonds and van der Waals interactions with the nitroalkene and Hantzsch ester. Subsequent publications further showcased the potential of octahedral chiral-at-metal complexes as inert templates for asymmetric catalysis.^22^ However, this approach does not represent asymmetric transition metal catalysis in the real sense and can be rather classified as asymmetric organocatalysis with an inert metal template. In this context, it is also worth mentioning the pioneering work by Fu and coworkers who used planar-chiral sandwich complexes for asymmetric nuclephilic catalysis.^23^ Although the metal does formally not serve as a stereogenic center, the metal is instrumental for generating planar chirality.

### 2.6. First examples of efficient asymmetric catalysis with chiral-at-metal complexes (2013–2014)

The development of chiral-at-metal catalysts capable of achieving high enantioselectivities represents a relatively recent advancement. In 2013, Hartung and Grubbs unveiled the chiral-at-ruthenium catalyst M8, designed for diastereo- and enantioselective ring-opening/cross-metathesis reactions (Fig. 2g).^24^ For instance, 1 mol% of Δ-M8 catalyzed the reaction of a norbornene derivative (12) with an excess of allyl acetate (11), providing diene 13 in 64% yield, with 95% *Z*-selectivity and 93% ee. It is worth noting that, in addition to the ruthenium center of complex M8, the ruthenium-bound carbon atom of the adamantyl group also acts as a stereogenic center. However, this stereogenicity is intrinsically tied to the metal–carbon bond, and the ligand becomes achiral when this bond is broken. In 2014, Meggers and coworkers introduced the first chiral-at-metal complex for enantioselective Lewis acid catalysis (Fig. 2h).^25^ For instance, the conjugate addition of indole to α,β-unsaturated acyl imidazole 14 afforded the addition product 15 with 97% yield and 96% ee using 1.0 mol% of Λ-IrO. This work introduced a general design strategy which ultimately highlighted the broad applicability and effectiveness of chiral-at-metal catalysts within asymmetric catalysis.

## 3. General design strategy for chiral-at-metal catalysts

### 3.1. Challenge in designing chiral-at-metal catalysts

The primary challenge in developing chiral-at-metal catalysts lies in ensuring that the metal center functions both as a configurationally stable stereocenter and as a reactive metal site. Unlike chiral metal complexes where chiral ligands often thermodynamically favor a single metal-centered configuration, chiral-at-metal complexes are prone to losing stereochemical information at the metal center due to ligand dissociation or isomerization.^9^

### 3.2. General design strategy

**Fig. 3:**
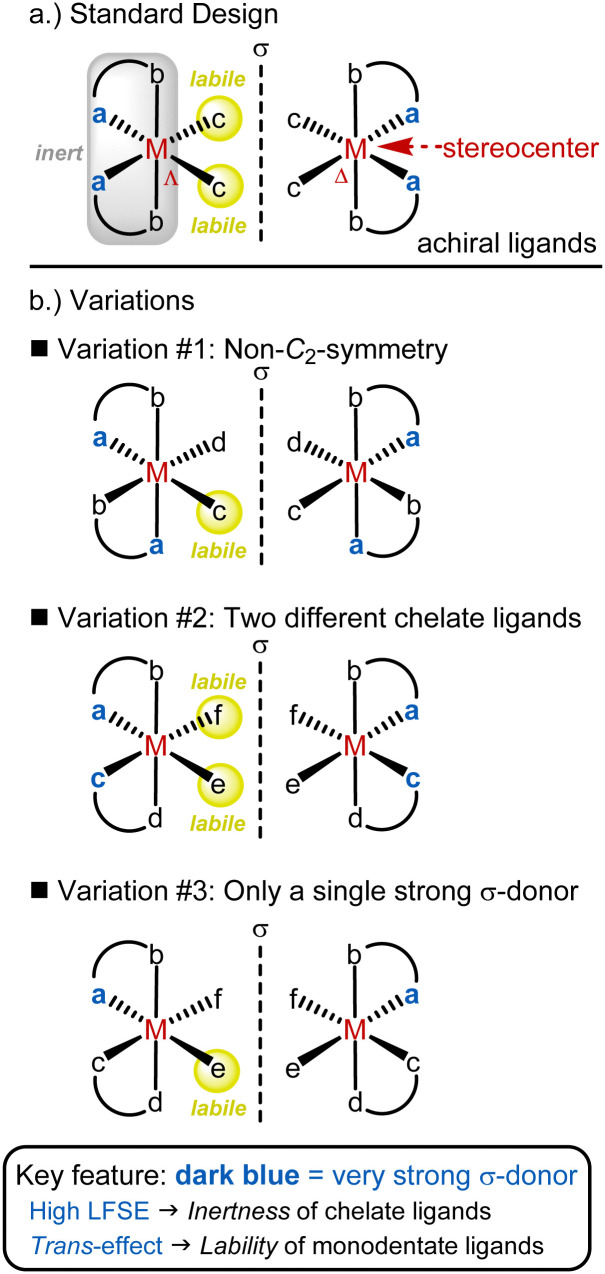
General design strategy for chiral-at-metal catalysts with an octahedral coordination geometry.

We developed a widely applicable structural framework for chiral-at-metal catalysts, centered around octahedral complexes with two *cis*-coordinated bidentate ligands and two monodentate ligands (Fig. 3).^26–28^ All ligands are achiral, and the overall chirality arises from the helical arrangement of the bidentate ligands, which formally creates a stereogenic metal center with either a Λ-configuration (left-handed helix) or Δ-configuration (right-handed helix). While these complexes are typically *C*_2_-symmetric, lower symmetry is also possible.

The foundation of the chiral-at-metal catalyst design involves incorporating inert bidentate ligands to establish and preserve the absolute stereochemistry at the metal center, alongside labile monodentate ligands that facilitate access to the metal center for substrates or reagents. This is achieved by leveraging three key effects: (1) the chelate effect, (2) high ligand field stabilization energy (LFSE),^29^ and (3) the *trans*-effect.^30^ While the chelate effect provides some extra stabilization for the bidentate ligands over the monodentate ones, this alone does not ensure inertness. In our experience, it is essential to combine the chelate effect with a strong ligand field, as the kinetic and thermodynamic properties of transition metal complexes are closely tied to the LFSE. This effect is particularly pronounced in octahedral low-spin 18-electron complexes. Additionally, tailored chelate ligands with strong σ-donating (raising the e_g_* orbital) and π-accepting (lowering the t_2g_ orbitals) properties are employed to maximize ligand field splitting. The strong σ-donating coordination sites also play a critical role by being positioned *trans* to the monodentate ligands, thereby destabilizing them *via* the *trans*-effect. Thus, the σ-donating sites serve a dual function: enhancing the inertness of the bidentate ligand framework while simultaneously labilizing the monodentate ligands. This design offers a blueprint for combining configurational stability of the metal stereocenter with a high lability of the monodentate ligands.

The most popular implementation of this design strategy is illustrated in Fig. 3a. In this *C*_2_-symmetric standard design, the two bidentate ligands are identical and their strongly σ-donating groups are positioned *trans* to the monodentate ligands (typically MeCN), which results in the strong labilization of these monodentate ligands. A modified version of this design (variation #1) involves a non-*C*_2_-symmetric diastereomer in which only one monodentate ligand is labile (Fig. 3b). Another variation (variation #2) introduces different bidentate ligands. Lastly, in variation #3, only one of the bidentate ligands includes a strongly σ-donating coordination site. As shown in Fig. 3, these different ligand arrangements directly influence the lability of the monodentate ligands, with only those positioned *trans* to a strongly σ-donating group being labile.

## 4. Asymmetric catalysis with chiral-at-metal complexes

### 4.1. Chiral-at-iridium catalysts

#### 4.1.1. Design of chiral-at-iridium catalysts

**Fig. 4:**
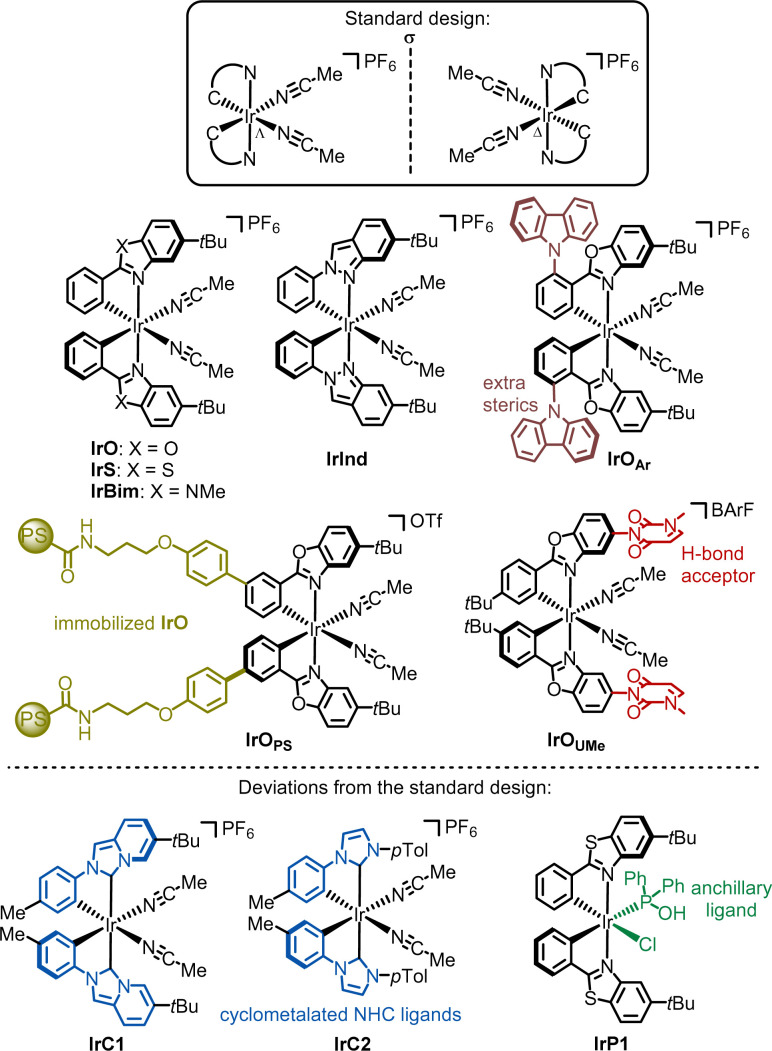
Overview of developed chiral-at-iridium catalysts.

Octahedral iridium(iii) complexes (18 valence electrons) are among the most inert transition metal complexes. Given that the preservation of the metal-centered configuration is crucial to the chiral-at-metal design, it is not surprising that some of the earliest chiral-at-metal catalysts were based on iridium in the +III oxidation state. The standard design, which is illustrated in Fig. 4 (framed complexes), consists of a central iridium atom which is cyclometalated by two phenyl-substituted aromatic heterocycles, with the octahedral coordination geometry completed by two labile acetonitrile ligands, resulting in an overall *C*_2_-symmetry.^26,31,32^ These cationic iridium complexes are typically used as hexafluorophosphate salts. The two inert *cis*-coordinated cyclometalating ligands create a stereogenic iridium center, which can adopt either a left-handed (Λ-configuration) or right-handed (Δ-configuration) helical topology. The strongly σ-donating phenyl ligands exert a significant *trans*-effect, which labilizes the two acetonitrile ligands. This is crucial for catalysis, where one or both monodentate ligands are substituted by a substrate or reagent, while the helically arranged inert cyclometalated ligands promote asymmetric induction. The catalysts IrO,^25^IrS,^33^IrBim,^34^ and IrInd^34^ all follow this standard design and feature cyclometalated ligands with N-coordinated bicyclic aromatic heterocycles, such as benzoxazole (IrO), benzothiazole (IrS), benzimidazole (IrBim), and benzindazole (IrInd). These heterocycles extend into the catalyst's active site, where their steric bulk is further enhanced by *tert*-butyl substituents on the phenyl groups, ensuring optimal asymmetric induction. The core scaffold has been further functionalized by adjusting steric demand and solubility (IrO_Ar_),^35^ incorporating an H-bond acceptor (IrO_UMe_),^36^ or attaching a chiral-at-iridium catalyst to a solid support (IrO_PS_).^37^

Deviations from the standard design have also been reported. For instance, IrC1^38^ and IrC2^39^ differ from the other chiral-at-iridium catalysts as they contain cyclometalated N-heterocyclic carbene (NHC) ligands. More recently, a bis-cyclometalated iridium catalyst featuring an anchillary phosphine oxide ligand (IrP1) was introduced.^40^

#### 4.1.2. Synthesis of chiral-at-iridium catalysts

Enantiomerically pure chiral-at-iridium catalysts are typically synthesized using a chiral-auxiliary strategy.^41^ In this approach, a racemic mixture of chiral-at-metal complexes is reacted with a chiral auxiliary ligand, transforming the racemate into a mixture of diastereomers. This allows for separation based on solubility or chromatographic differences. Finally, the chiral auxiliary ligand is removed to produce enantiomerically pure chiral-at-metal catalysts. A crucial feature of the chiral auxiliary ligands is their tunable binding strength, which can be increased by deprotonation or decreased by reprotonation, enabling reversible coordination of the chiral auxiliary ligands.^42,43^

**Fig. 5:**
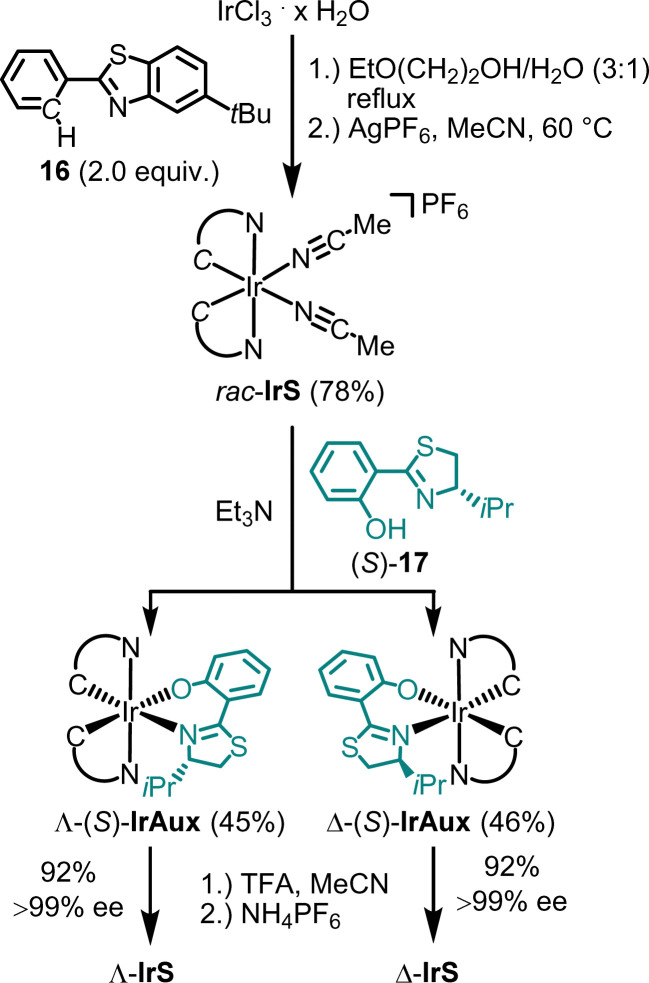
Chiral auxiliary-mediated synthesis of enantiomerically pure catalysts Λ-IrS and Δ-IrS.

The synthesis of Λ- and Δ-IrS is shown as an example in Fig. 5.^44^ Accordingly, starting from IrCl_3_, the reaction with 2-phenylbenzothiazole (16) followed by the reaction with AgPF_6_ in MeCN provides *rac*-IrS (78%) in a diastereoselective manner as a racemate. The subsequent reaction of *rac*-IrS with the chiral thiazoline (*S*)-17 then produces the two diastereomeric complexes Λ-(*S*)-IrAux (45%) and Λ-(*S*)-IrAux (46%), which are separated using standard silica gel chromatography. These are then converted to virtually enantiopure Λ-IrS and Δ-IrS (92%, each >99% ee) by replacing the chiral auxiliary thiozoline ligand with two acetonitrile ligands through treatment with TFA in MeCN followed by NH_4_PF_6_ to obtain the hexafluorophosphate salts.

#### 4.1.3. Chiral-at-iridium catalysis applications

##### 4.1.3.1. Lewis acid catalysis

**Fig. 6:**
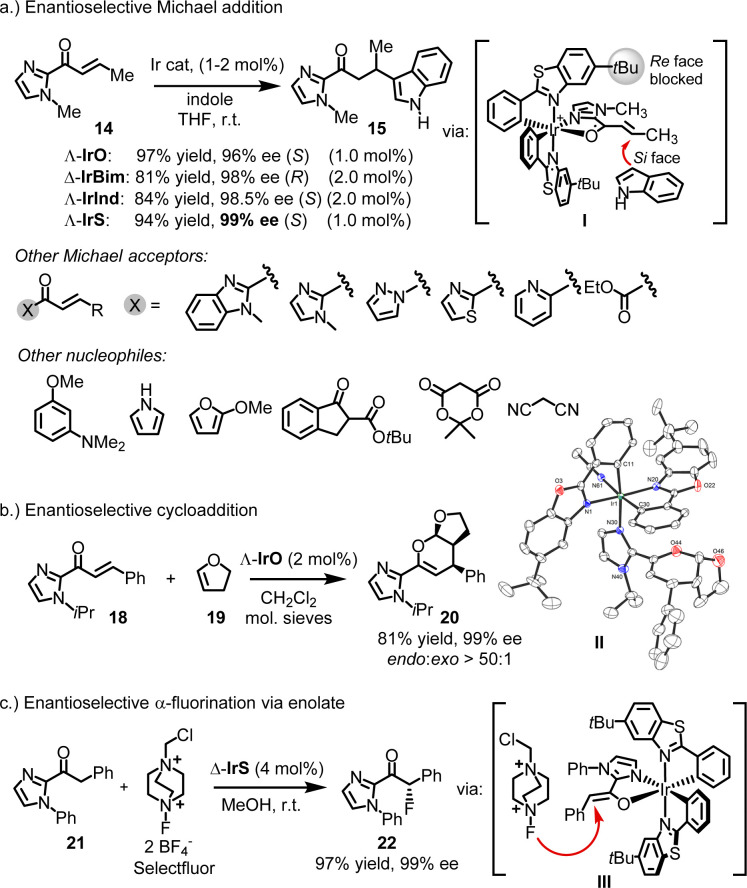
Chiral Lewis acid catalysis with chiral-at-iridium catalysts. This typically involves substrate activation through two-point binding by the iridium catalyst.

In many of the reported cases, the chiral-at-iridium complexes function as chiral Lewis acids.^26,31,32^ What sets them apart from the wide variety of available chiral Lewis acid catalysts is their exceptional rigidity, which typically results in very high enantiomeric excess values. For instance, IrO, IrS, IrBim, and IrInd have been shown to act as chiral Lewis acid catalysts for the enantioselective conjugate addition of indoles to α,β-unsaturated 2-acyl imidazoles.^25,34,45^ In a specific example, the reaction of unsaturated 2-acyl imidazole 14 with indole, catalyzed by 1 or 2 mol% of chiral-at-iridium catalysts, yielded the Michael adduct 15 in 81–97% yield with 96–99% ee (Fig. 6a). Mechanistically, the bidentate N,O-coordination of the 2-acyl imidazole to the iridium center activates it toward attack by the nucleophilic indole (I). The chiral bis-cyclometalated iridium fragment effectively blocks the *Re*-face of the prochiral alkene (in the case of metal-centered Λ-configuration), directing the indole to the *Si*-face and providing strong asymmetric induction. In this and many other reported Michael reactions, using a variety of different Michael acceptors and nucleophiles, the benzothiazole-based catalysts Λ- and Δ-IrS consistently offer the best enantioselectivities. This can be attributed to the long C–S bond lengths in the benzothiazole, which position the *tert*-butyl groups somewhat closer to the catalytic site, enhancing steric hindrance. This often results in near-perfect asymmetric induction, with many examples achieving enantioselectivities of 99% ee.

There are instances where IrO outperforms IrS, such as in the hetero-Diels–Alder reaction between enone 18 and 1,2-dihydrofuran (19), producing the bicyclic product 20 with an 81% yield, excellent diastereoselectivity (*endo *: *exo* > 50 : 1), and 99% ee (Fig. 6b).^45^ A crystal structure of the product coordinated to the catalyst could represent an intermediate in the catalytic cycle (II).

Chiral-at-iridium complexes have also been employed in enolate chemistry. Xu reported remarkably high enantioselectivities in an enantioselective α-fluorination of 2-acyl imidazole 21 using Selectfluor, yielding the fluorinated product 22 with 97% yield and 99% ee.^46^ This reaction likely proceeds *via* an iridium enolate intermediate (III) (Fig. 6c).

Other reactions reported to be catalyzed by chiral-at-iridium complexes include asymmetric Nazarov cyclizations^47^ and the kinetic resolution of epoxides^35^ using CO_2_ to produce cyclic carbonates.

##### 4.1.3.2. Asymmetric transfer hydrogenation

**Fig. 7:**
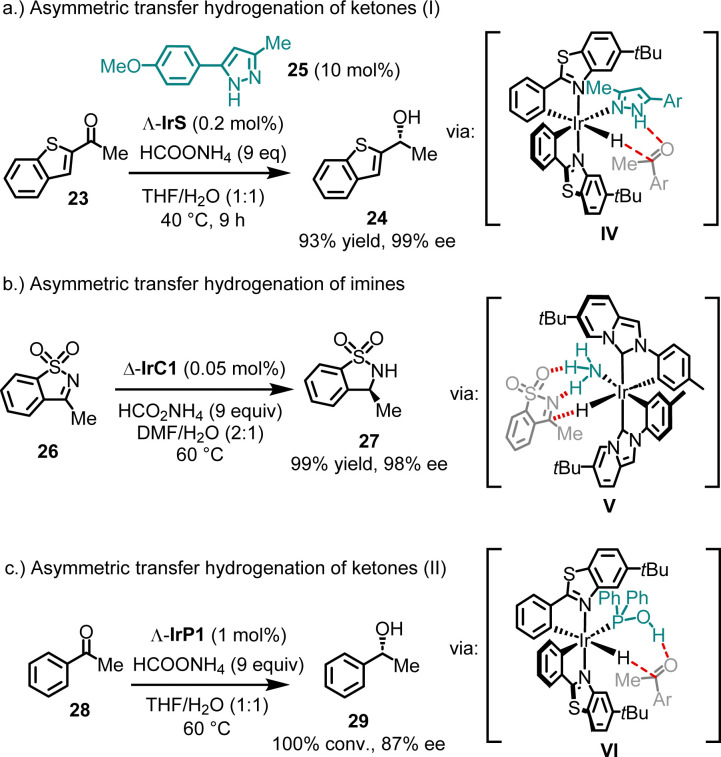
Asymmetric transfer hydrogenation with chiral-at-iridium complexes.

In addition to conventional chiral Lewis acid catalysis, bis-cyclometalated chiral-at-iridium complexes have been demonstrated to catalyze asymmetric hydrogenations^48^ and transfer hydrogenations.^38,40,49,50^ Mechanistically, these examples share the feature of an ancillary monodentate ligand coordinating to the iridium catalyst, facilitating hydrogen bonding with the substrate (see intermediates IV–VI in Fig. 7). For example, 2-acetyl benzothiophene (23) was reduced to its alcohol 24 with 93% yield and 99% ee, using ammonium formate as the reducing agent and only 0.2 mol% of the catalyst Λ-IrS (Fig. 7a).^49^ Remarkably, quantitative conversion with 98% ee was achieved even at a catalyst loading as low as 0.005 mol%. It is important to note that the presence of a pyrazole co-ligand (*e.g.*25) is essential for this catalysis, as it binds to the iridium catalyst, while reaction with ammonium formate generates an iridium hydride intermediate. This allows a Noyori–Ikariya-type transition state^51^ (IV), resulting in the concerted transfer of a hydride to the carbonyl carbon and a proton to the carbonyl oxygen.

In a related example, IrC1, bearing cyclometalated NHC ligands, was shown to catalyze the enantioselective reduction of sulfonylimines (Fig. 7b).^38^ For instance, using just 0.05 mol% of Δ-IrC1 with ammonium formate as the reducing agent, sulfonylimine 26 was converted to sultam 27 with 99% yield and 98% ee. Mechanistic studies indicated that in this system, NH_3_ acts as the ancillary ligand (V).

In contrast to these cases, where the ancillary ligand coordinates *in situ*, Pazos and Freixa reported that the complex IrP1, with a secondary phosphine oxide coordinated in its tautomeric hydroxy form, catalyzes the asymmetric transfer hydrogenation of acetophenone (28) to produce alcohol 29 with complete conversion and 87% ee (Fig. 7c).^40^ Here, the hydroxy group of the coordinated phosphine is proposed to form a hydrogen bond with the ketone in the transition state (VI).

##### 4.1.3.3. Asymmetric photoredox catalysis

**Fig. 8:**
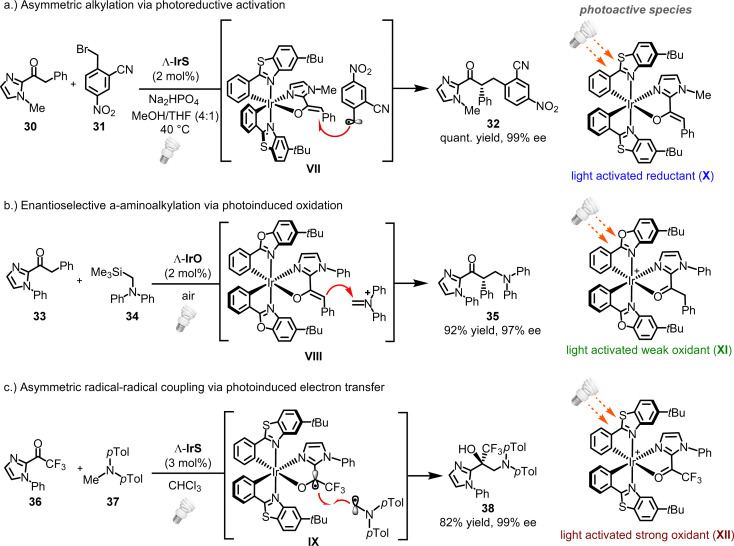
Asymmetric photoredox catalysis with chiral-at-iridium complexes.

What truly distinguishes this class of chiral-at-iridium catalysts, however, is their capability to catalyze asymmetric photoreactions.^26^ Bis-cyclometalated iridium complexes are widely employed as photoredox catalysts or photosensitizers in contemporary organic photochemistry.^52^ In the realm of asymmetric photochemistry, these complexes are often paired with a chiral catalyst to achieve dual catalysis.^53^ Notably, we have documented several instances where IrO or IrS act as the sole catalyst for asymmetric photoredox catalysis.^33,54–57^ For example, visible light-induced radical α-alkylation of 2-acyl imidazole 30 with the electron-deficient benzyl bromide 31 afforded 32 quantitatively with 99% ee (*via* reaction of enolate intermediate VII with an electrophilic radical) (Fig. 8a),^33^ while the aminoalkylation of 2-acyl imidazole 33 with α-silylamine 34 under air afforded 35 with 92% yield and 97% ee (*via* the reaction of enolate intermediated VIII with an iminium ion) (Fig. 8b).^54^ In another case, IrS facilitated the reaction of trifluoromethylketones (*e.g.*36) with tertiary amines (*e.g.*37) to produce chiral 1,2-aminoalcohols (*e.g.*38) with 82% yield and 99% ee, through a photoredox-catalyzed radical–radical cross-coupling (intermediate IX) (Fig. 8c).^57^ In these asymmetric photoredox reactions, the chiral iridium catalyst functions both as a chiral Lewis acid and as a photocatalyst, following substrate binding.

In all reported asymmetric photoreactions catalyzed by chiral-at-iridium complexes, it is noteworthy that the photoactive iridium species are generated *in situ* through substrate coordination, occasionally followed by deprotonation. The photochemical properties of these intermediates then govern the reaction pathways. The neutral iridium enolate intermediate (X) (Fig. 8a) functions as a strong photoactivated reductant. In contrast, the cationic iridium complex (XI), formed upon binding to a 2-acyl imidazole, acts as a weak photoactive oxidant (Fig. 8b). Meanwhile, coordination with more electron-deficient 2-trifluoroacetyl imidazoles (complex XII) generates a significantly stronger light-activated oxidant (Fig. 8c).

In conclusion, it is striking that the structurally simple chiral-at-metal iridium complexes, IrO and IrS, are capable of catalyzing such intricate visible-light-driven transformations, where asymmetric catalysis closely cooperates with photoredox chemistry. Furthermore, it is important to highlight that, in these reactions, the metal center assumes multiple roles: it serves as the sole source of chirality, acts as the Lewis acid center, and is a crucial component of the photoactive species generated *in situ*.

### 4.2. Chiral-at-rhodium catalysts

#### 4.2.1. Overview of developed chiral-at-rhodium catalysts

**Fig. 9:**
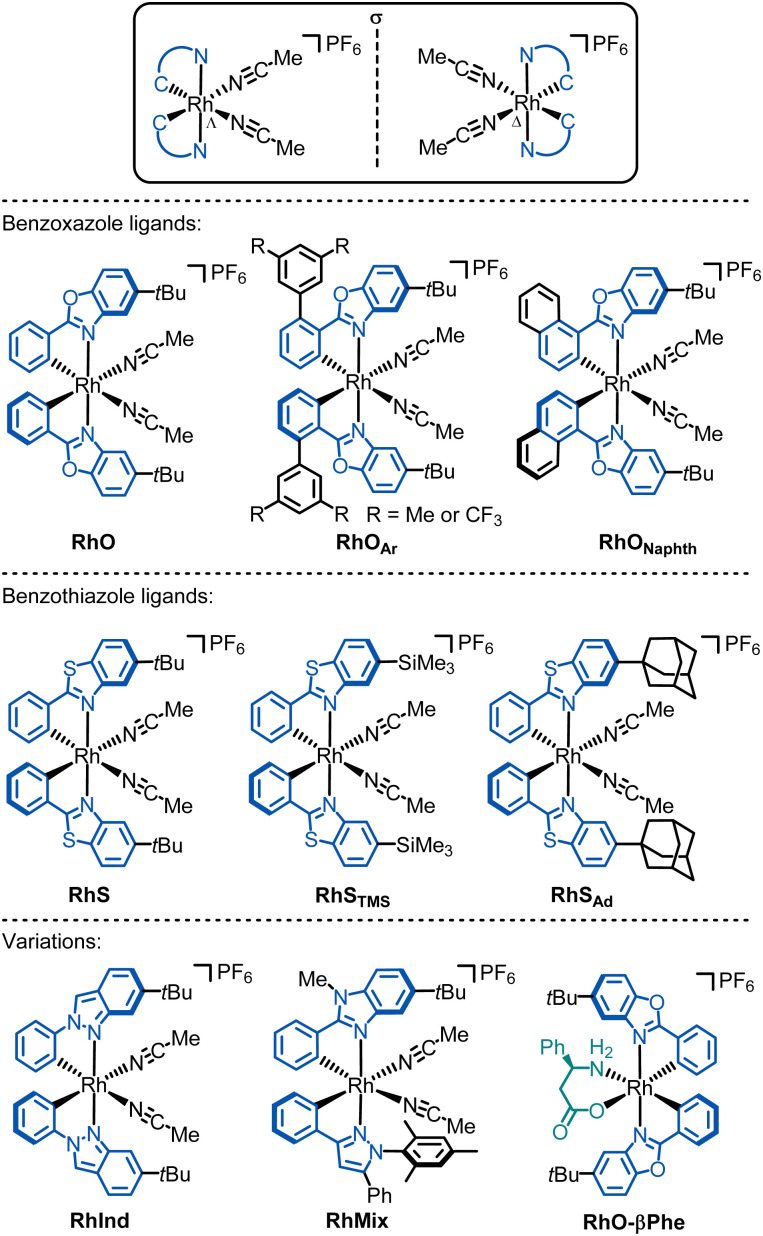
Overview of developed chiral-at-rhodium catalysts.

Fig. 9 provides an overview of the chiral-at-rhodium catalysts developed to date, which have been utilized in a wide range of applications in asymmetric catalysis.^58–129^ In 2015, our group reported the first rhodium catalyst featuring exclusively metal-centered chirality.^58^ In this catalyst, RhO, which is the lighter congener of IrO, the rhodium is cyclometalated in a propeller-like, *C*_2_-symmetrical arrangement by two 5-*tert*-butyl-2-phenylbenzoxazole ligands, creating a stereogenic rhodium center with either a left-handed (Λ) or right-handed (Δ) metal-centered configuration, resulting in a helical overall topology. The cyclometalated ligands are configurationally inert, maintaining the stereochemistry of the rhodium center. The octahedral coordination sphere is completed by two labile acetonitrile ligands, which are necessary for catalytic activity by allowing substrate or reagent binding upon the dissociation of one or both acetonitrile ligands. A hexafluorophosphate anion balances the charge of the monocationic rhodium complex.

Since the cyclometalating ligands and their substitution patterns significantly influence catalytic performance and enantioselectivity, several other rhodium catalysts have been developed. Kang reported RhO derivatives with 3,5-Me_2_Ph or 3,5-(CF_3_)_2_Ph moieties on the phenyl groups of the phenylbenzoxazole ligands (RhO_Ar_).^99,100^ In another modification, Kang and Du replaced the cyclometalated phenyl moiety with a naphthyl (RhO_Naphth_).^110^

We further developed the chiral-at-Rh catalyst RhS, the lighter analogue of IrS, which features cyclometalated phenylbenzothiazole ligands instead of phenylbenzoxazoles.^61^

**Fig. 10:**
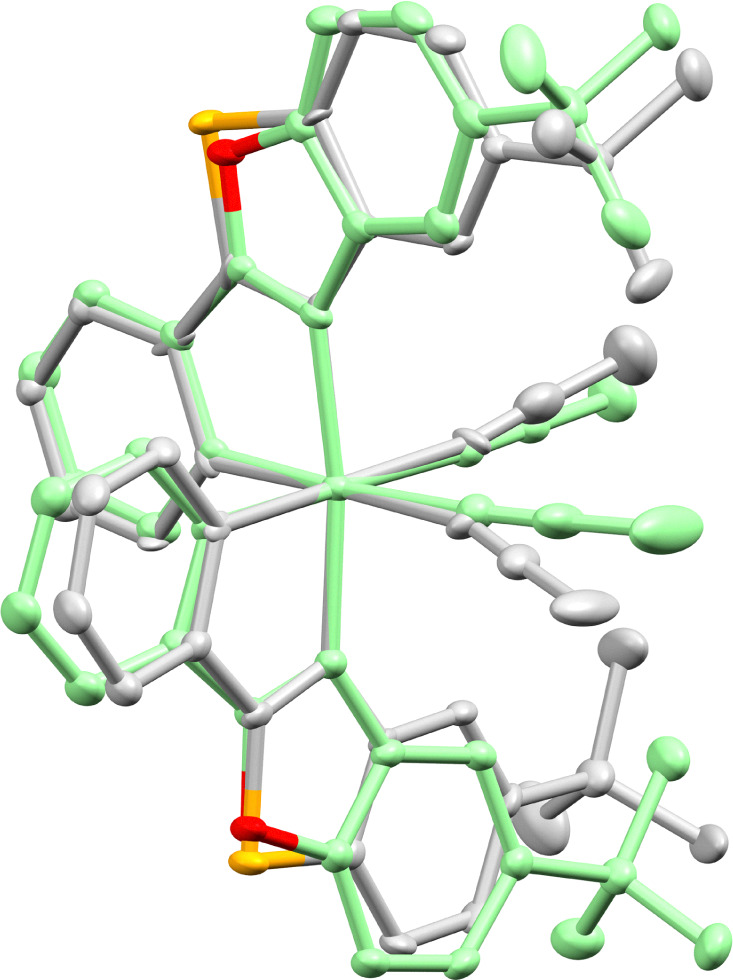
Superimposed crystal structure of RhS (grey, CCDC 1455731) and RhO (green, CCDC 1027145). Reproduced from ref. 61 with permission from The Royal Society of Chemistry, copyright 2016.

Importantly, the benzothiazole catalyst RhS often offers better enantioselectivities than its benzoxazole counterpart RhO. This can be attributed to the longer C–S bonds in the benzothiazole of RhS compared to the C–O bonds in the benzoxazole of RhO, bringing the *tert*-butyl groups of RhS closer to the active site (near the two labile acetonitrile ligands) and enhancing asymmetric induction (see Fig. 10 for superimposed RhS and RhO). The steric crowding at the active site can be further enhanced by substituting the *tert*-butyl groups with more bulky adamantyl (RhS_Ad_)^77^ or trimethylsilyl (RhS_TMS_)^85^ groups. Another variant we developed is RhInd,^87,93^ the lighter congener of IrInd, where rhodium is cyclometalated by two phenylindazole ligands. We also introduced chiral-at-rhodium catalysts with two different cyclometalating ligands, such as RhMix, which incorporates both phenylbenzimidazole and phenylpyrazole ligands.^86^ Finally, the pre-catalyst RhO-βPhe disintegrates *in situ* to form a chiral-at-rhodium Lewis acid and β-phenylalanine to perform chiral Lewis acid/enamine co-catalysis.^65^

All these chiral-at-rhodium complexes are air- and moisture-stable, tolerate silica gel chromatography, and remain fully configurationally stable in solution at room temperature.

#### 4.2.2. Synthesis of chiral-at-rhodium catalysts

**Fig. 11:**
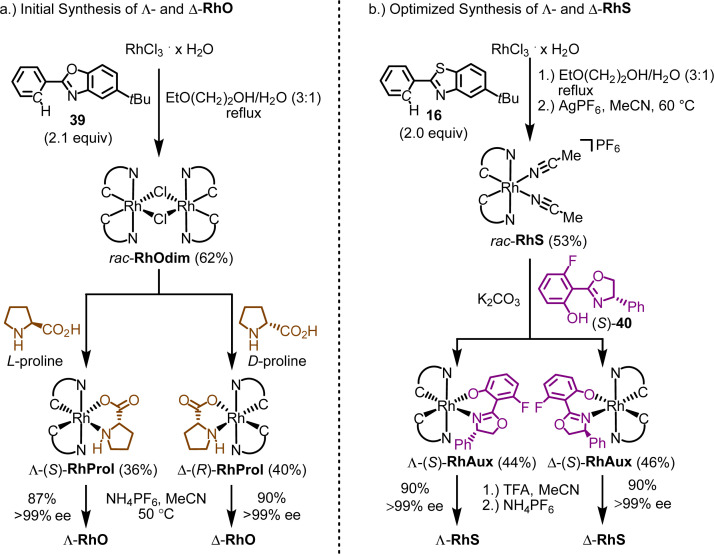
Chiral auxiliary-mediated synthesis of enantiomerically pure chiral-at-rhodium catalysts. (a) Λ- and Δ-RhO using l- and d-proline as chiral auxiliary ligands from the chiral pool. (b) Λ- and Δ-RhS using a fluorinated salicyloxazoline ligand.

The initial synthesis of enantiomerically pure RhO used proline as a chiral auxiliary from the chiral pool (Fig. 11a).^58^ Accordingly, the reaction of RhCl_3_ hydrate with 5-*tert*-butyl-2-phenylbenzoxazole (39) in a 3 : 1 mixture of 2-ethoxyethanol and water under reflux afforded the rhodium dimer complex *rac*-RhOdim (62%). A subsequent reaction of *rac*-RhOdim with d-proline provided a mixture of diastereomers, Λ-(*R*)-RhProl and Δ-(*R*)-RhProl, which could not be separated by chromatography due to the limited stability of the complexes. However, we discovered that Δ-(*R*)-RhProl could be easily isolated in high purity with a 40% yield by washing the diastereomeric mixture with a CH_2_Cl_2_/diethyl ether solution, effectively exploiting the solubility difference between the two diastereomers. In an analogous fashion, the mirror-imaged complex Λ-(*S*)-RhProl can be synthesized using l-proline as the chiral auxiliary (36% yield). Exposure of the individual diastereomers to the weak acid NH_4_PF_6_ in MeCN at 50 °C, to labilize the prolinato ligand by protonation, resulted in a replacement of proline with two acetonitriles, thus affording Λ-RhO and Δ-RhO as single enantiomers (>99% ee). Thus, the abundant amino acid proline served as a convenient chiral auxiliary to generate enantiomerically pure chiral-at-rhodium catalysts Λ- and Δ-RhO.

Finding a chiral-auxiliary-mediated method to generate enantiomerically pure RhS proved more challenging, as all initially tested chiral auxiliaries failed to afford intermediate rhodium auxiliary complexes with distinct solubility differences and lacked the stability required for separation by silica gel chromatography. Finally, a fluorinated salicyloxazoline turned out to be suitable (Fig. 11b).^61^ The auxiliary-mediated synthesis starts with rhodium trichloride hydrate which is first reacted with 5-*tert*-butyl-2-phenylbenzothiazole (16) followed by treatment with AgPF_6_ in MeCN to provide racemic RhS (73%). The complex *rac*-RhS is then reacted with the monofluorinated salicyloxazoline (*S*)-40 to provide a diastereomeric mixture of Λ-(*S*)-RhAux and Δ-(*S*)-RhAux. In contrast to RhProl, both diastereomers are sufficiently stable to be resolved into the single diastereomers (46% each) by silica gel chromatography. Alternatively, they can also be resolved based on their different solubilities in EtOH, or a combination of chromatography and exploiting solubility differences. Finally, starting with Λ-(*S*)-RhAux or Δ-(*S*)-RhAux, a TFA induced replacement of the coordinated auxiliary ligand with two acetonitriles under retention of configuration followed by treatment with NH_4_PF_6_ affords the individual enantiomers Λ-RhS (90%) and Δ-RhS (90%). A very detailed synthetic protocol is available.^44^

**Fig. 12:**
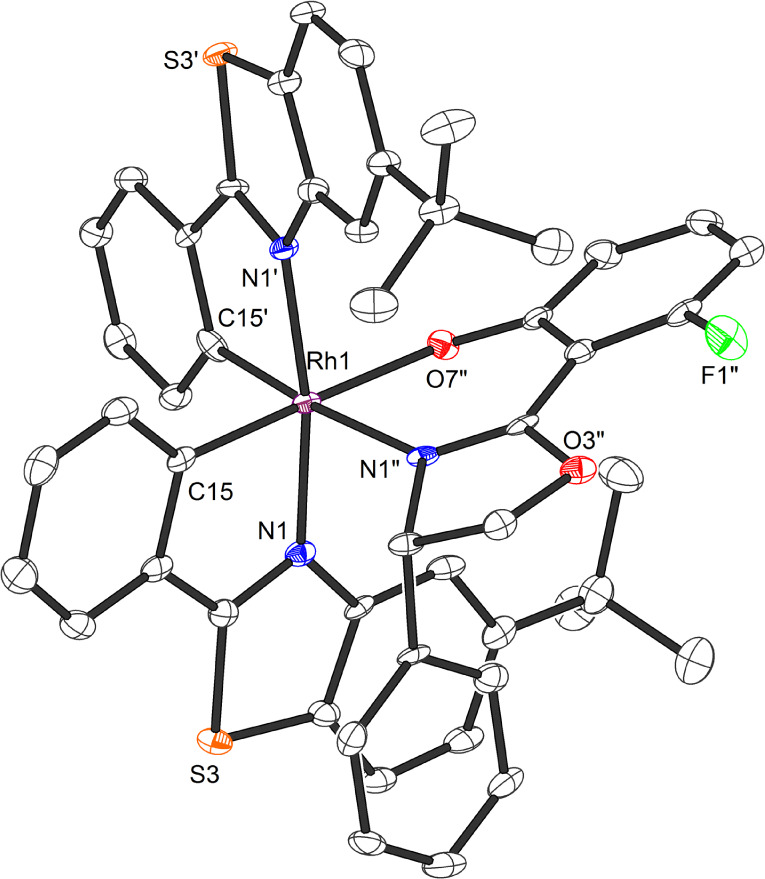
Crystal structure of the chiral auxiliary chiral-at-rhodium complex Λ-(*S*)-RhAux (CCDC 1455732). Reproduced from ref. 61 with permission from The Royal Society of Chemistry, copyright 2016.

A crucial element of this auxiliary-mediated synthesis is the fluorinated salicyloxazoline ligand (*S*)-40 which was originally introduced by Ceroni and co-workers.^130^ The fluorine renders the coordinated phenolate less basic and thus stabilizes towards Lewis acid activation (such as silica gel) while the phenyl substituent of the oxazoline moiety undergoes π-stacking with the adjacent benzothiazole ligand (Fig. 12). These two aspects lead to an important improvement of the stability of the auxiliary complex so it can be handled and chromatographed without decomposition.

#### 4.2.3. Chiral-at-rhodium catalysis applications

Bis-cyclometalated chiral-at-rhodium complexes are excellent chiral Lewis acids. The large body of published work on chiral-at-rhodium catalysis demonstrates that, for most applications, chiral-at-rhodium complexes are superior chiral Lewis acid catalysts compared to their isostructural chiral-at-iridium counterparts. This can be pinpointed to kinetic effects, specifically a much faster rate of ligand exchange, which is at least three orders of magnitude higher.^64^ Since ligand exchange is a critical step in any catalytic cycle (*e.g.* substrate coordination and product dissociation), this becomes especially significant in radical reactions, where the typically short lifetime of an intermediate radical demands a high turnover frequency in the catalytic cycle. Bis-cyclometalated chiral-at-rhodium complexes have also been extensively applied to asymmetric photocatalysis^27^ and used for catalytic asymmetric electrochemistry^85,94^ and even photoelectrochemistry.^95^

**Fig. 13:**
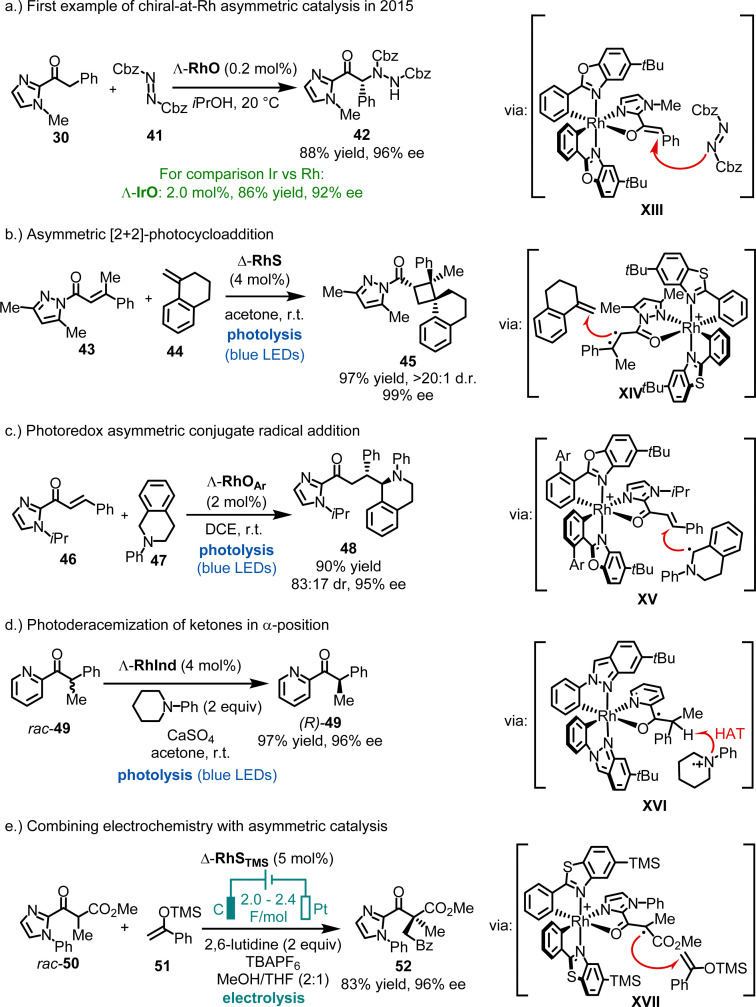
Catalytic applications of chiral-at-rhodium catalysts.

The first examples of chiral-at-rhodium catalysis were reported by our group in 2015 (Fig. 13a).^58^ For instance, Λ-RhO, at a catalyst loading of just 0.2 mol%, was shown to catalyze the enantioselective α-amination of 2-acyl imidazole 30 with dibenzyl azodicarboxylate (41), affording product 42 in 88% yield and 96% ee. In comparison, the heavier congener Λ-IrO required ten times the catalyst loading to achieve 86% yield and 92% ee. Mechanistically, the reaction is believed to proceed through a rhodium enolate intermediate (XIII), where the *Si*-face of the enolate's α-carbon is shielded by one of the *tert*-butyl groups, leading to an efficient asymmetric induction during the reaction of the rhodium enolate with the azodicarboxylate electrophile. Building on this initial work, chiral-at-rhodium complexes such as RhO, RhS, and their derivatives have been established by our group,^58–95^ as well as by Kang, Du, Su, Gong, Li and Xu,^96–116,118–129^ and others^89,92,117^ as highly versatile chiral Lewis acid catalysts for a broad range of reactions. In most catalytic processes, the substrate is activated through bidentate binding to the electrophilic rhodium center (*e.g.* see proposed catalytic intermediates XIII–XVII). Common substrates include 2-acyl imidazoles, *N*-acyl pyrazoles, 2-acyl pyridines, 1,3-dicarbonyl compounds, among others.

Additionally, bis-cyclometalated rhodium complexes have proven to be excellent catalysts for asymmetric photochemistry by Meggers,^27^ Kang,^102^ and Alemán,^117^ whether employed in dual catalysis or as single catalysts. For example, we demonstrated highly enantioselective RhS-catalyzed [2+2] photocycloadditions under direct visible-light excitation (43 + 44 → 45) (Fig. 13b),^73^ while Kang reported a visible-light-induced asymmetric conjugate radical addition using RhO_Ar_ (Ar = 3,5-(CF_3_)_2_Ph) (46 + 47 → 48) (Fig. 13c).^102^ Recently, a bis-cyclometalated rhodium indazole complex (RhInd) was shown to catalyze the efficient α-photoderacemization of pyridylketone 49 (Fig. 13d).^93^ The manifold applications of bis-cyclometalated chiral-at-rhodium complexes for asymmetric photocatalysis was reviewed recently.^27^

Additionally, bis-cyclometalated rhodium complexes have proven to be effective catalysts for integrating electrochemistry with asymmetric catalysis.^85,94^ For instance, Meggers recently reported that RhS_TMS_ catalyzes the oxidative cross-coupling of racemic 2-acyl imidazole *rac*-50 with silyl enol ether 51, providing a route to non-racemic 1,4-dicarbonyl compound 52 in 83% yield and 96% ee (Fig. 13e).^85^

In summary, since the first report in 2015,^58^ numerous studies have demonstrated the versatility of bis-cyclometalated chiral-at-rhodium complexes in asymmetric catalysis, particularly in reactions where the substrate binds *via* a two-point interaction. This versatility stems from their strong Lewis acid activity, in conjunction with photoactivity (triggered *in situ* upon substrate binding) and high chemical stability, making them suitable for a wide range of reaction types, including photoreactions, radical reactions, and redox processes.

### 4.3. Chiral-at-ruthenium and chiral-at-osmium catalysts

#### 4.3.1. Overview of developed chiral-at-ruthenium catalysts

**Fig. 14:**
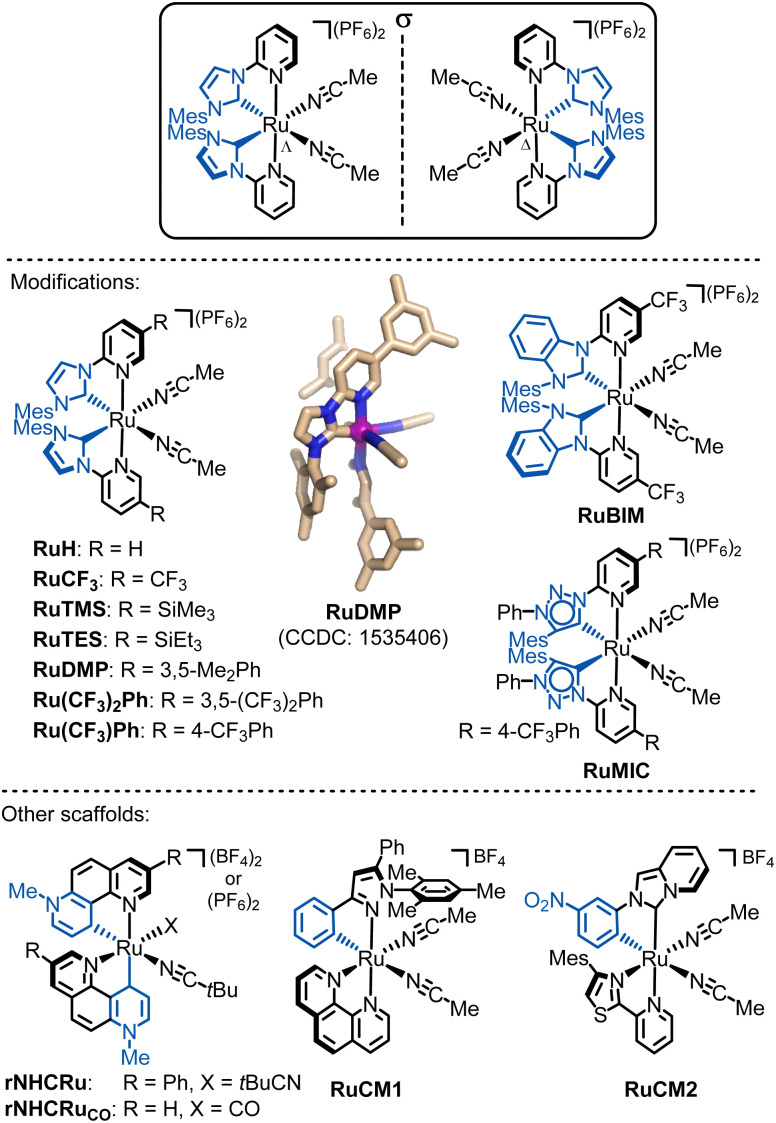
Overview of developed chiral-at-ruthenium catalyts. The shown crystal structure of RuDMP is reproduced from ref. 131 with permission from the American Chemical Society, copyright 2017.

A widely successful chiral-at-ruthenium catalyst design is illustrated in Fig. 14.^28,131–144^ The structure features ruthenium(ii) coordinated in a *cis*-arrangement by two chelating N-(2-pyridyl)-substituted N-heterocyclic carbene (PyNHC) ligands, along with two acetonitrile ligands. The *C*_2_-symmetric dicationic complex is commonly paired with two hexafluorophosphate anions, which ensures its solubility in standard organic solvents. The complex is chiral, despite its achiral ligands, due to the stereogenicity of the metal center. The absolute configuration of the metal center is determined by the helical twist of the two PyNHC ligands, resulting in either a Λ (left-handed) or Δ (right-handed) configuration. The complex [Ru(PyNHC)_2_(MeCN)_2_] exhibits high constitutional and configurational stability for the PyNHC ligands, while the acetonitriles are highly labile. The high inertness of the Ru(PyNHC)_2_ core can be attributed to the electronic properties of the PyNHC ligands, which combine a strong σ-donating NHC group with a σ-donating and π-accepting pyridyl group, maximizing ligand field stabilization energy and thus enhancing both the kinetic and thermodynamic properties of the octahedral complex. Additionally, the strong σ-donating NHC groups weaken the coordinated acetonitrile ligands through the *trans*-effect. Inter-ligand stacking interactions between the mesityl groups of the NHC and pyridyl moieties further contribute to the robustness of the catalyst (see X-ray structure of RuDMP in Fig. 14). This design enables the catalyst to perform well in asymmetric catalysis, even at temperatures exceeding 100 °C. Moreover, the electronic and steric properties of the scaffold can be tuned by adding substituents to the pyridyl (see RuCF_3_, RuTMS, RuTES, RuDMP, Ru(CF_3_)_2_Ph, Ru(CF_3_)Ph) or NHC components (see RuBIM), creating catalysts optimized for various asymmetric reactions. The scaffold's robustness and ability to catalyze stereocontrolled nitrene-mediated C(sp^3^)–H aminations makes it particularly valuable. Recently, a new derivative featuring 1,2,3-triazol-5-ylidene mesoionic carbenes in place of imidazol-2-ylidenes has been introduced (RuMIC), significantly enhancing the reactivity of ruthenium complexes in enantioselective intramolecular C(sp^3^)–H aminations of aliphatic azides to produce chiral pyrrolidines.^145^

In a different design, our group in collaboration with Houk developed a chiral-at-ruthenium catalyst where ruthenium is cyclometalated by two 7-methyl-1,7-phenanthrolinium heterocycles in a non-*C*_2_-symmetric manner, forming chelating pyridylidene remote N-heterocyclic carbene ligands (rNHCs) (rNHCRu).^146^ Due to the lack of *C*_2_-symmetry, the two coordinated nitrile ligands are not equivalent. The strong σ-donating pyridylidene ligand renders the *trans*-positioned acetonitrile more labile, as evidenced by an elongated coordination bond. This catalyst demonstrated remarkable activity in the intramolecular C(sp^3^)–H amidation of 1,4,2-dioxazol-5-ones, leading to the formation of chiral γ-lactams. However, the *C*_2_-symmetric diastereomer promotes the undesired Curtius rearrangement. This example highlights the significance of both the relative stereochemistry (*C*_2_-symmetric *vs.* non-*C*_2_ symmetric diastereomer) and the absolute metal-centered stereochemistry (Λ *versus* Δ) in determining catalytic activity and enantioselectivity.

In addition to NHC, MIC, or rNHC groups as strongly σ-donating chelate ligands, incorporating even stronger σ-donating ligands, such as cyclometalating ligands that form metal-carbon σ-bonds, can increase the electron density at the ruthenium center, potentially yielding chiral-at-ruthenium catalysts with unique catalytic properties. For instance, the catalyst RuCM1,^147^ which features a cyclometalating phenylpyrazole ligand and coordinated 1,10-phenanthroline, is highly effective in converting diazoketones to chiral flavanones. Meanwhile, RuCM2,^148^ which features a cyclometalated N-(3-nitrophenyl)-imidazo[1,5-*a*]pyridinylidene ligand along with a bidentate 4-mesityl-2-(pyridin-2-yl)thiazole, catalyzes the enantioselective intramolecular cyclopropanation of *trans*-cinnamyl diazoacetate and an alkenyl diazoketone, leading to the formation of bicyclic cyclopropanes.

#### 4.3.2. Synthesis of chiral-at-Ru catalysts

**Fig. 15:**
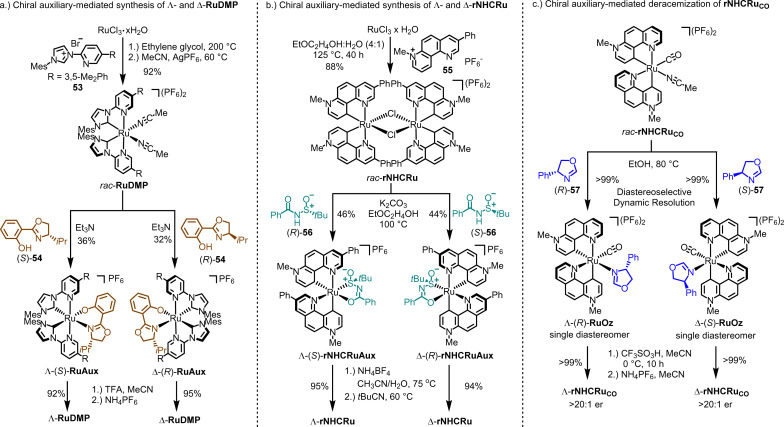
Chiral auxiliary-mediated synthesis of chiral-at-ruthenium catalysts. (a) Chiral auxiliary-mediated synthesis of Λ- and Δ-RuDMP. (b) Chiral auxiliary-mediated synthesis of Λ- and Δ-rNHCRu. (c) Synthesis of a non-racemic chiral-at-ruthenium catalyst Λ- and Δ-rNHCRu_CO_ by chiral-auxiliary-mediated deracemization.

The synthetic route for producing enantiomerically pure *C*_2_-symmetric chiral-at-ruthenium complexes is outlined in Fig. 15a, using the preparation of Λ- and Δ-RuDMP as an example.^131^ Accordingly, heating a mixture of ruthenium trichloride hydrate and pyridyl imidazolium salt 53 in ethylene glycol at 200 °C, followed by treatment with AgPF_6_ in MeCN at 60 °C, yields the racemic complex *rac*-RuDMP. Reacting *rac*-RuDMP with the chiral salicyloxazoline auxiliary ligand (*S*)-54 in the presence of triethylamine affords the single diastereomer Λ-(*S*)-RuAux. Interestingly, the Δ-(*S*)-RuAux diastereomer does not form, likely due to steric clashes between the *S*-configured auxiliary ligand and the right-handed helical structure of the ruthenium Δ-enantiomer. In the next step, Λ-(*S*)-RuAux undergoes stereospecific exchange of the auxiliary ligand with two acetonitriles to afford enantiopure Λ-RuDMP. This transformation is achieved by treating the complex with trifluoroacetic acid (TFA) and ammonium hexafluorophosphate in acetonitrile, followed by purification through silica gel chromatography. By using (*R*)-54 as the chiral auxiliary ligand, enantiopure Δ-RuDMP can be synthesized through the same process. These chiral-at-ruthenium complexes are obtained in enantiomerically pure form and exhibit high constitutional and configurational stability, showing no signs of decomposition or racemization even after heating at 60 °C in THF for 72 hours.

The synthesis of enantiomerically pure Λ- and Δ-rNHCRu follows a similar overall strategy, but involves a different type of chiral auxiliaries and includes an unexpected isomerization.^146^ As depicted in Fig. 15b, reacting RuCl_3_ hydrate with 1,7-phenanthrolinium salt 55 in a 4 : 1 mixture of 2-ethoxyethanol and water at 125 °C results in the racemic chloro-bridged dimer complex *rac*-rNHCRu. Both ruthenium atoms are cyclometalated in a *C*_2_-symmetric manner by two 1,7-phenanthroline ligands, which can be described as chelating pyridyl pyridylidene ligands. In the subsequent step, the racemic mixture is reacted with either (*R*)- or (*S*)-*N*-benzoyl-*tert*-butanesulfinamide (56) in the presence of K_2_CO_3_, resulting in the formation of the single diastereomers, Λ-(*S*)-rNHCRuAux or Δ-(*R*)-rNHCRuAux, respectively. Remarkably, during the formation of these *N*-sulfinylcarboximidate complexes, an unexpected yet critical isomerization of the chelating pyridylidene ligands takes place. Finally, treatment with the weak acid NH_4_BF_4_, followed by reaction with pivalonitrile, yields the Λ-rNHCRu and Δ-rNHCRu catalysts.

All the methods discussed so far for generating chiral-at-metal catalysts have relied on chiral-auxiliary-ligand-mediated resolutions of racemic mixtures of the catalysts or its precursors. However, when the metal center exhibits lower configurational stability, such as at elevated temperature, alternative strategies become viable, allowing for the conversion of a racemic mixture into a single enantiomer of the catalyst. In line with this, we recently developed an auxiliary-mediated deracemization protocol for the synthesis of a non-*C*_2_-symmetric chiral-at-ruthenium catalyst (Fig. 15c).^149^ This catalyst features two cyclometalated 7-methyl-1,7-phenanthrolinium heterocycles, a CO ligand, and a labile MeCN ligand (rNHCRu_CO_, Fig. 14). When the monodentate chiral oxazoline ligand (*R*)-57 is coordinated in ethanol at 80 °C, the racemic mixture of the complex is converted into a single diastereomer, Λ-(*R*)-RuOz, with a quantitative yield through selective precipitation from the solution. At this elevated temperature, the two enantiomers of rNHCRu_CO_ are in equilibrium, and coordination with (*R*)-57 induces selective precipitation of one diastereomer. After the oxazoline ligand is removed under acidic conditions, enantiomerically pure Λ-rNHCRu_CO_ (>20 : 1 er) is obtained. The opposite enantiomer, Δ-rNHCRu_CO_, can be isolated using the chiral auxiliary ligand (*S*)-57 in an analogous fashion. While rNHCRu_CO_ is not an especially versatile catalyst, it was shown to catalyze the enantioselective conversion of 1,2,4-dioxazol-5-ones to their chiral γ-lactams.^149^

#### 4.3.3. Chiral-at-Ru catalysis applications

##### 4.3.3.1. Initial catalysis reports

**Fig. 16:**
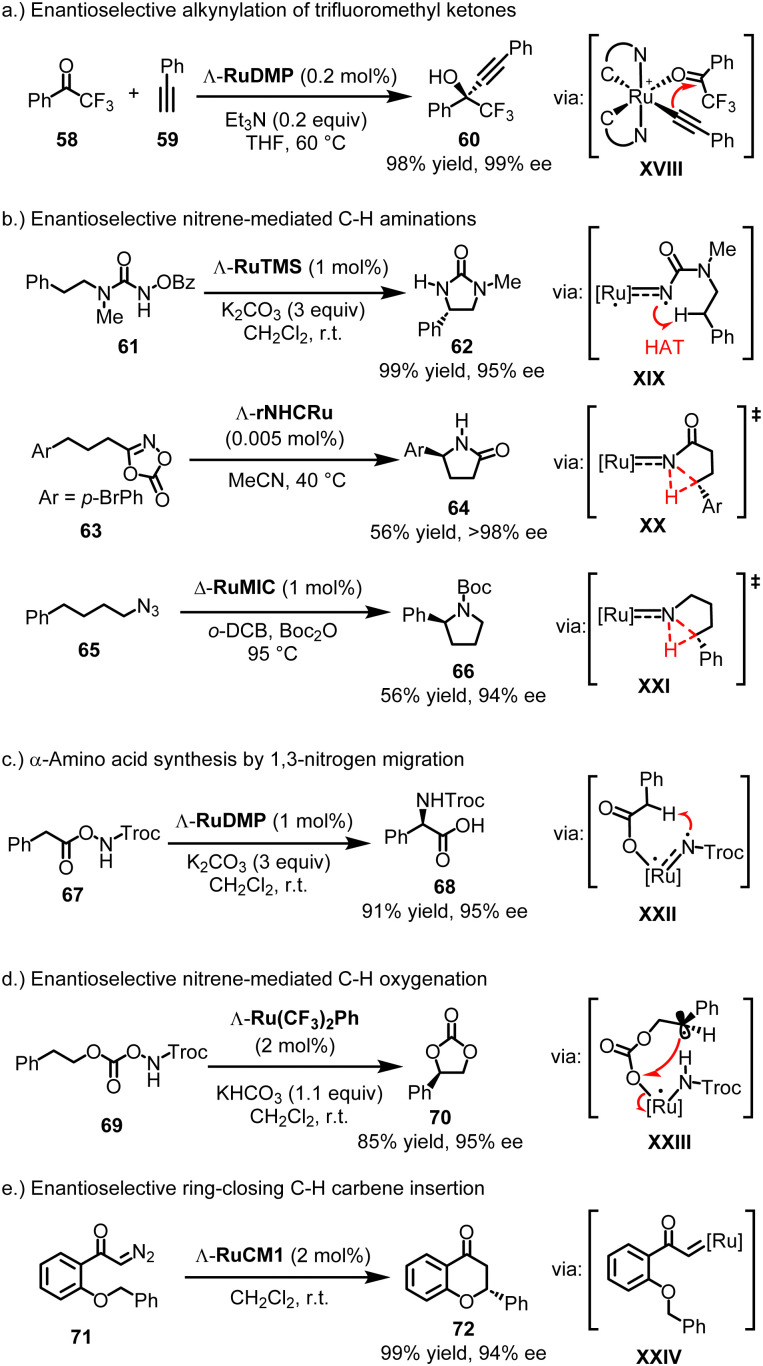
Catalytic applications of chiral-at-ruthenium catalysts.

The propeller-shaped Ru(PyNHC)_2_ catalysts were first employed in enantioselective alkynylation reactions involving trifluoromethyl ketones. For instance, at a catalyst loading of just 0.2 mol%, Λ-RuDMP efficiently catalyzes the alkynylation of 2,2,2-trifluoroacetophenone (58) with phenylacetylene (59), using catalytic amounts of the base Et_3_N, providing propargylic alcohol 60 with 98% yield and 99% ee (Fig. 16a).^131^ This approach was later utilized in the synthesis of key chiral intermediates for the AIDS drug Efavirenz.^132^ Mechanistically, experiments and DFT calculations by Chen and Houk are consistent with a mechanism in which the catalytic cycle proceeds *via* a ruthenium acetylide intermediate (XVIII), where the acetylide adds through an inner-sphere transfer to the ruthenium-bound trifluoromethyl ketone.^133^ The high rigidity of the propeller-shaped geometry contributes to the high enantioselectivity.

##### 4.3.3.2. Nitrene-mediated C−H aminations

Subsequently, it was discovered that the Ru(PyNHC)_2_ complexes exhibit remarkable catalytic efficiency and stereocontrol in a large variety of nitrene-mediated asymmetric C(sp^3^)–H amination reactions.^28^ For example, Λ-RuTMS (1 mol%) catalyzes the ring-closing C–H amination of *N*-benzoyloxyurea 61, affording 2-imidazolidinone 62 in 99% yield and 95% ee, likely through a triplet ruthenium nitrene intermediate (XIX), followed by a 1,5-hydrogen atom transfer (HAT) and subsequent C–N bond formation (Fig. 16b).^138^ In contrast, the rNHC complex rNHCRu displays less versatility compared to the Ru(PyNHC)_2_ complexes. However, it shows exceptional catalytic performance in the ring-closing C–H amidation of dioxazolones.^146^ For instance, dioxazolone 63 was converted to chiral γ-lactam 64 in 56% yield and >98% ee using merely 0.005 mol% of Λ-rNHCRu. DFT calculations suggest a mechanism involving intramolecular C–H nitrene insertion *via* a ruthenium *N*-acylnitrene intermediate (XX). Regarding mesoionic chiral-at-ruthenium catalysts, the complex Δ-RuMIC (1 mol%) has been shown to catalyze the challenging ring-closing C–H amination of aliphatic azide 65, forming pyrrolidine 66 in 56% yield and 94% ee, likely through a ruthenium nitrene C–H insertion (XXI).^145^ Maybe most interesting is the discovery that the chiral-at-ruthenium Ru(PyNHC)_2_ complexes catalyze a novel 1,3-migratory nitrene C(sp^3^)–H insertion, which constitutes a highly straightforward and economic α-amino acid synthesis.^143^ For example, Λ-RuDMP (1 mol%) catalyzes the conversion of azanyl ester 67 to amino acid 68 in 91% yield and 95% ee (likely *via***XXII**) (Fig. 16c).

##### 4.3.3.3. Nitrene-mediated C−H oxygenations

In addition to C–H aminations and amidations, chiral-at-ruthenium complexes have been reported to mediate uncommon nitrene-driven intramolecular C(sp^3^)–H oxygenations.^137,142^ For instance, Λ-Ru(CF_3_)_2_Ph (2 mol%) catalyzes the conversion of azanyl carbonate 69 to cyclic carbonate 70 with an 85% yield and 95% ee (likely *via***XXIII**) (Fig. 16d).^142^

##### 4.3.3.4. Carbene C–H insertions

Finally, cyclometalated chiral-at-ruthenium catalysts have been reported to catalyze carbene chemistry such as enantioselective ring-closing C(sp^3^)–H carbene insertions.^147,148^ For example, Λ-RuCM1 (2 mol%) catalyzes the conversion of diazoketone 71 to flavanone 72 with a 99% yield and 94% ee (likely *via***XXIV**) (Fig. 16e).^147^

To summarize this section on chiral-at-ruthenium complexes, the *cis*-coordination of bidentate ligands to ruthenium results in a helical arrangement of the chelate ligands, creating a stereogenic metal center. Given the extensive development of ruthenium coordination chemistry and the numerous methods available for the stepwise or simultaneous introduction of ligands, a wide range of chiral-at-ruthenium catalysts have been created. Crucially, at least one of the bidentate ligands contains a strongly σ-donating group, such as a conventional NHC, a remote NHC, a mesoionic carbene, or a cyclometalating ligand. Among the various chiral-at-ruthenium catalysts, the *C*_2_-symmetric complexes [Ru(PyNHC)_2_(MeCN)_2_]_2_ (PF_6_)_2_ stand out due to their chemical robustness, high asymmetric induction, and strong catalytic performance, particularly in nitrene-mediated asymmetric reactions. This catalyst framework has been recently reviewed.^28^

#### 4.3.4. Chiral-at-osmium catalysis

**Fig. 17:**
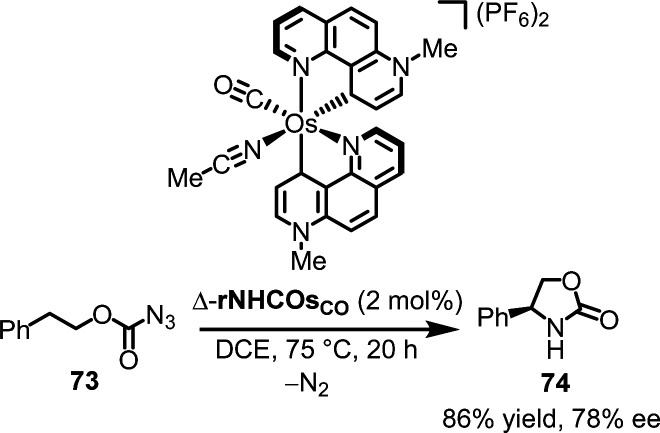
First example of a chiral-at-osmium catalysis.

The chiral-at-osmium catalyst rNHCOs_CO_, which is isostructural to rNHCRu_CO_, was recently reported by us (Fig. 17).^150^ However, because coordinative bonds to osmium are significantly less labile than those to ruthenium, single enantiomers of this complex were obtained using conventional chiral-auxiliary-mediated resolution in contrast to the auxiliary-mediated reracemization applied to the isostructural ruthenium catalyst rNHCRu_CO_. The chiral-at-osmium catalyst rNHCOs_CO_ was shown to promote the ring-closing C(sp^3^)–H amidation of azidoformates. For instance, using 2.0 mol% of Δ-rNHCOs_CO_ at 75 °C, azidoformate 73 was converted into 2-oxazolidinone 74 with an isolated yield of 86% and 78% ee.

### 4.4. Chiral-at-iron catalysts

**Fig. 18:**
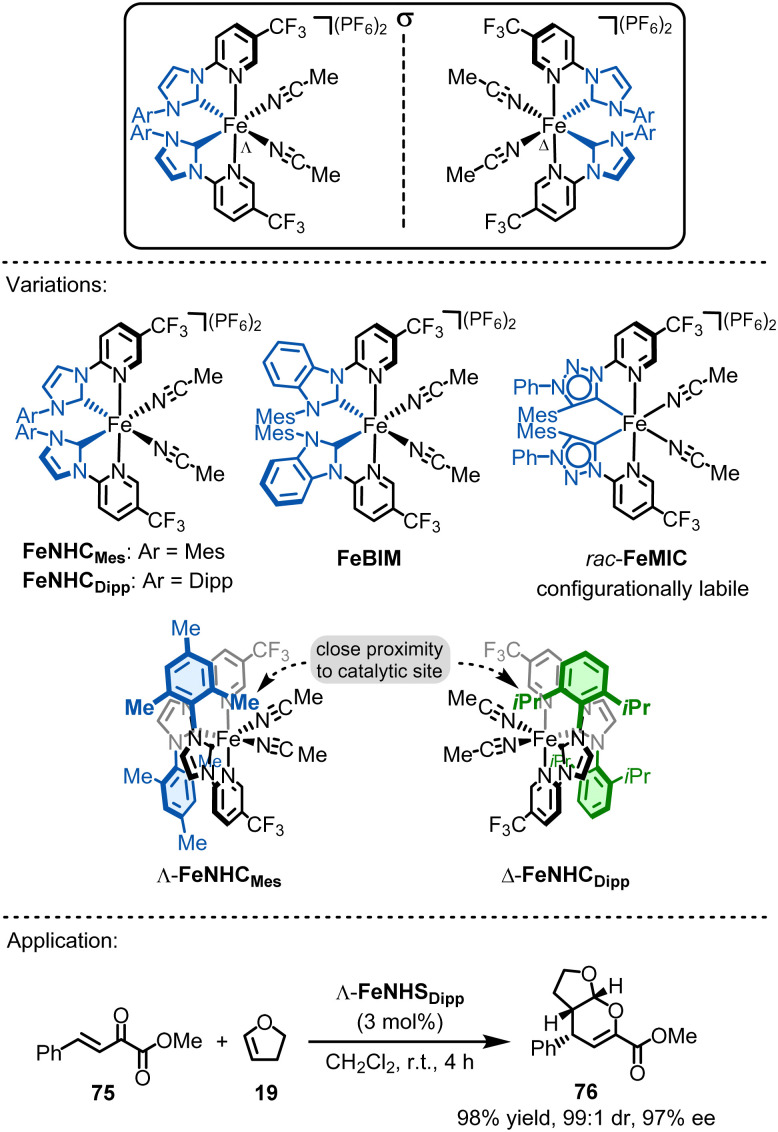
Chiral-at-iron catalysis.

The development of reactive chiral-at-metal catalysts using earth-abundant metals is highly challenging due to the high lability of coordination bonds with 3d metals and the crucial need for a metal stereocenter that is configurationally stable. In 2019, our group reported the first chiral-at-iron catalyst, showcasing how ligand design can significantly influence both configurational stability and ligand lability.^151^ The catalyst employs the same ligand framework as related Ru(PyNHC)_2_ complexes.^28^ Specifically, iron is coordinated by two chelating N-(2-pyridyl)-substituted N-heterocyclic carbenes (PyNHC) in a *C*_2_-symmetrical arrangement, forming a helical structure with the iron center adopting either the Λ (left-handed) or Δ (right-handed) stereogenic configuration (Fig. 18). The coordination is completed by two acetonitrile molecules, resulting in an overall octahedral geometry. The dicationic iron complexes are typically used as hexafluorophosphate salts.

Similar to the related chiral-at-ruthenium catalysts,^28^ the strongly σ-donating NHC moieties, combined with the σ-donating and π-accepting pyridyl ligands, provide a strong ligand field. This increases the stability of the helical arrangement of the two bidentate ligands, while the *trans*-effect of the σ-donating NHC ligands ensures high lability of the two acetonitrile ligands. Interestingly, it was shown that the CF_3_ groups have a positive effect on the stability of the iron complexes. Furthermore, the configurational stability depends on the nature of the σ-donating NHC ligands. For instance, replacing the imidazol-2-ylidene carbene groups of FeNHC_Mes_ with slightly more π-accepting benzimidazol-2-ylidenes (FeBIM)^152^ increases configurational robustness. Conversely, replacing them with more strongly σ-donating 1,2,3-triazol-5-ylidene mesoionic carbenes (FeMIC)^153^ completely eliminates configurational stability. Therefore, precise ligand design is essential for creating configurationally robust chiral-at-iron catalysts.

These chiral-at-iron complexes have proven to be suitable chiral Lewis acid catalysts for asymmetric hetero-Diels–Alder reactions. Notably, asymmetric induction in these reactions can be enhanced by replacing the 2,4,6-trimethylphenyl (Mes) groups at the NHC ligands (FeNHC_Mes_) with bulkier 2,6-diisopropylphenyl (Dipp) groups (FeNHC_Dipp_).^154^ These larger moieties interact directly with the catalytic site, influencing the asymmetric induction in inverse electron demand hetero-Diels–Alder reactions (see Fig. 18). For example, Λ-FeNHC_Dipp_ (3 mol%) catalyzes the reaction between the α,β-unsaturated α-ketoester 75 and 2,3-dihydrofuran (19) to afford 3,4-dihydro-2*H*-pyran 76 in 98% yield with 99 : 1 dr and 97% ee.

### 4.5. Other chiral-at-metal catalyst designs

**Fig. 19:**
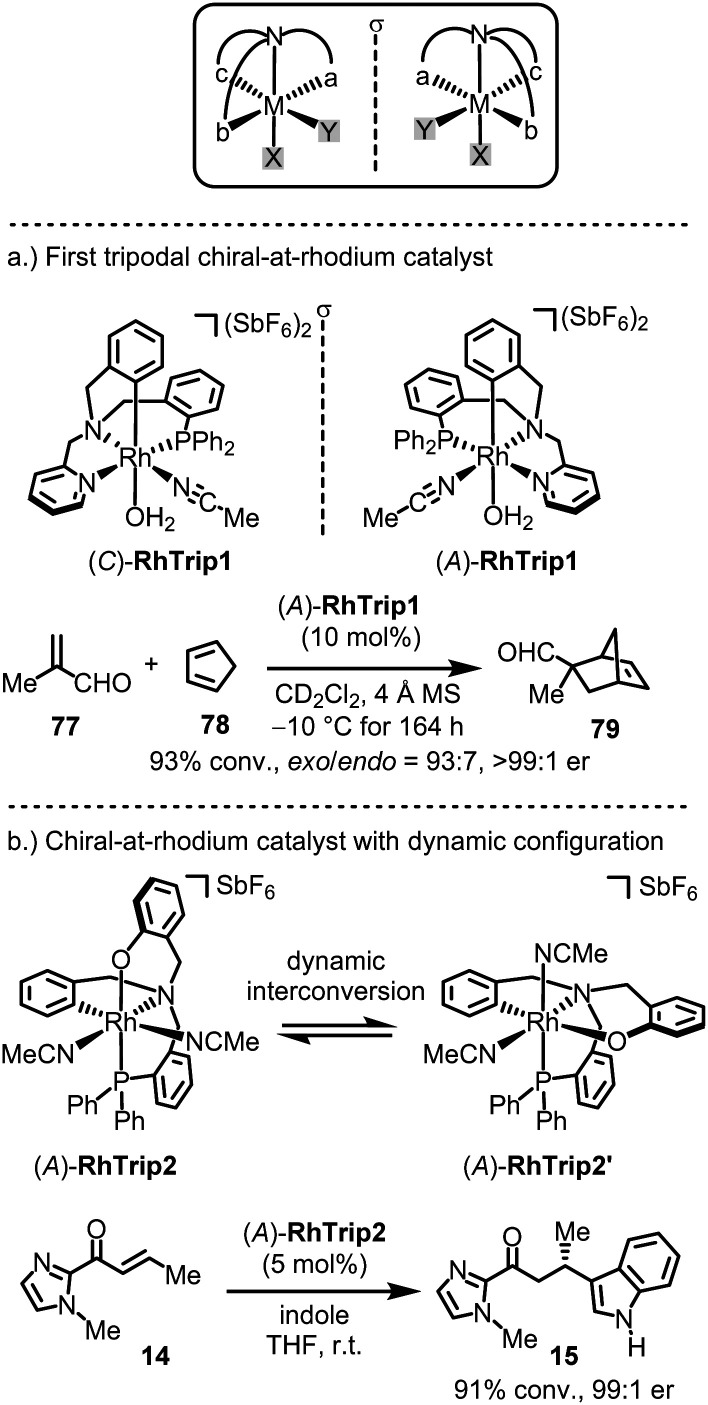
Chiral-at-rhodium catalyts based on a tripodal ligand design.

A few chiral-at-metal catalysts have been reported that differ from the general design discussed in the previous section. Notably, Rodríguez, Passarelli and Carmona introduced an intriguing chiral-at-metal catalyst design through the use of a tripodal, tetradentate ligand featuring three distinct arms branching from a central coordinating amine moiety (Fig. 19a).^155–162^ Enantiomerically pure chiral-at-rhodium complexes (*C*)-RhTrip1 (clockwise absolute metal-centered configuration) and (*A*)-RhTrip1 (anticlockwise absolute metal-centered configuration) were synthesized using the amino acids (*S*)- or (*R*)-phenylglycine, respectively.^157^ The authors demonstrated that (*A*)-RhTrip1 functions as a chiral Lewis acid, efficiently catalyzing the Diels–Alder reaction between methacrolein (77) and cyclopentadiene (78) to provide the norbornene derivative 79 with high enantioselectivity (>99 : 1 er). DFT calculations support a mechanism in which the chiral Lewis acid (*A*)-RhTrip1 forms a well-defined chiral pocket. Methacrolein is then activated by coordinating to the aldehyde oxygen, while the diphenylphosphino arm of the tripodal ligand shields the *Si*-face, leaving the *Re*-face open for interaction with cyclopentadiene. Such tripodal chiral-at-rhodium complexes can also catalyze the enantioselective alkylation of indoles with nitrostyrenes,^159^ and 1,3-dipolar cycloadditions.^160^

Recently, the same group introduced a modified tripodal chiral-at-rhodium catalyst, RhTrip2, where the pyridyl moiety is substituted by a phenoxy group (Fig. 19b).^162^ Notably, RhTrip2 undergoes dynamic interconversion to its diastereomer, RhTrip2′, without racemization. Additionally, RhTrip2 was shown to catalyze the conjugate addition of indole (14 → 15) with high enantiomeric excess.

**Fig. 20:**
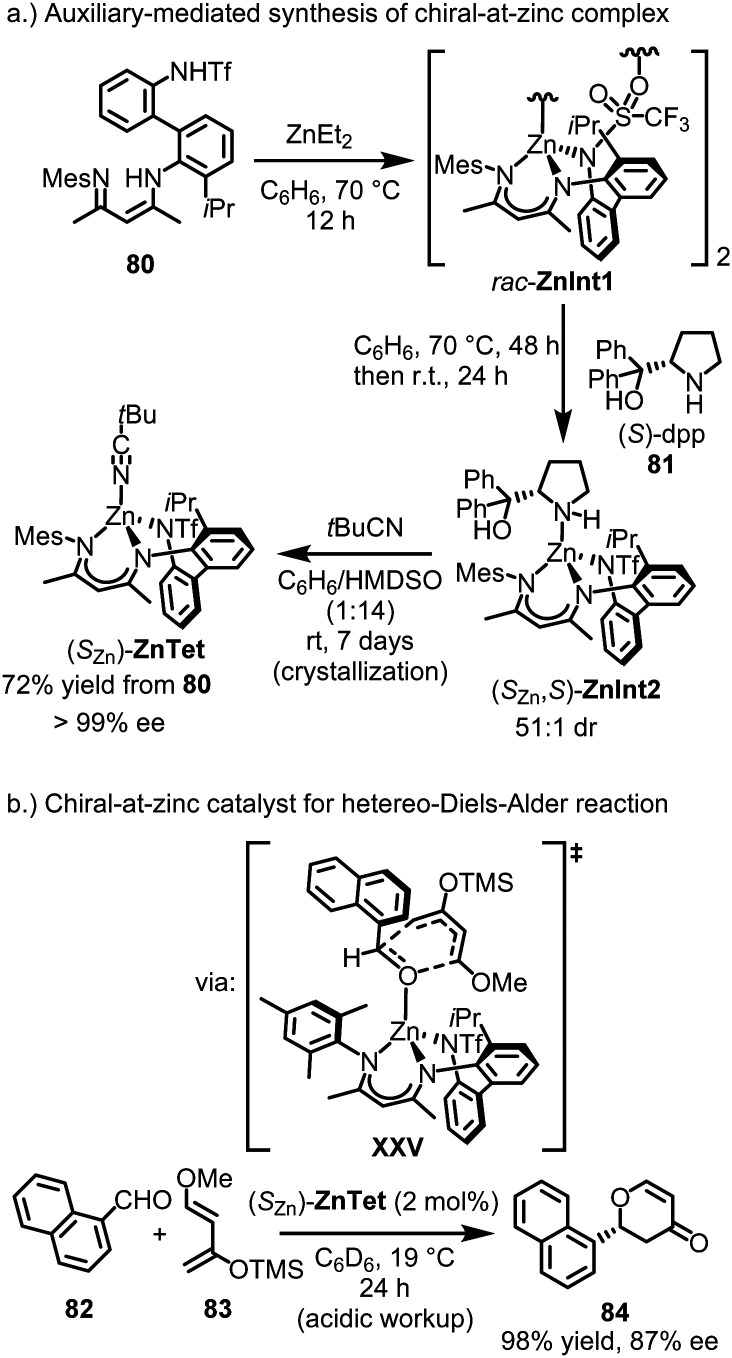
Chiral-at-zinc catalyt with tetrahedral coordination sphere.

Shionoya's group reported the only known example of a tetrahedral, chiral-at-metal, asymmetric catalyst to date.^163^ They successfully synthesized a remarkably stable tetrahedral chiral-at-zinc complex, which was shown to catalyze an asymmetric oxa-Diels–Alder reaction by serving as a chiral Lewis acid. This was achieved through the use of the tailored tridentate ligand 80 (Fig. 20a). Upon reaction of ligand 80 with ZnEt_2_, the dimeric zinc complex *rac*-ZnInt1 was formed as a racemic mixture. The racemic complex was then treated with the chiral pyrrolidine (*S*)-dpp (81), serving as a monodentate ligand and leading to the formation of the complex (*S*_Zn_,*S*)-ZnInt2 with a high diastereomeric ratio of 51 : 1 dr. NMR analysis revealed that, following coordination of (*S*)-dpp, the racemic zinc complex initially generated a nearly equal mixture of diastereomers, (*S*_Zn_,*S*)- and (*R*_Zn_,*S*)-ZnInt2. Over time, the mixture converted to the more thermodynamically stable (*S*_Zn_,*S*)-diastereomer, suggesting a stereoinversion at the zinc center. The chiral auxiliary (*S*)-dpp was removed by treatment with *t*BuCN at room temperature for 7 days, resulting in the crystallization of (*S*_Zn_)-ZnTet as a single enantiomer (>99% ee), with a 72% yield starting from ligand 80. The final complex, (*S*_Zn_)-ZnTet, contains a stereogenic zinc center with four distinct coordinating groups. The configurational stability of (*S*_Zn_)-ZnTet is likely influenced by the axial chirality of the biphenyl group in the backbone of the tridentate ligand, which becomes locked into a single conformation upon coordination with zinc. Interestingly, Shionoya recently also demonstrated that a related non-racemic nickel(ii) complex could be obtained by spontaneous resolution to form conglomerate crystals, without the need for any chiral sources.^164^

(*S*_Zn_)-ZnTet (2 mol%) was shown to catalyze the oxa-Diels–Alder reaction between 1-naphthaldehyde (82) and the Danishefsky diene (83), affording dihydropyranone 84 in 98% yield with 87% ee (Fig. 20b). The authors obtained a crystal structure in which 1-naphthaldehyde is coordinated to the (racemic) zinc catalyst by replacing the labile *t*BuCN ligand. This structure suggests the mechanism of asymmetric induction, where the bulky mesityl group stacks with 1-naphthaldehyde, blocking the *Si*-face and forcing the diene to approach the carbonyl group from the *Re*-face in the transition state (XXV).

## 5. Summary and outlook

Alfred Werner first demonstrated metal-centered chirality over a century ago. However, until recently, asymmetric catalysis using chiral transition metal complexes has primarily depended on chiral ligands. Our group introduced a simple and general strategy that combines inert and labile ligands, enabling the metal center to function both as a stable metal stereocenter and as a reactive site for catalysis. Strong σ-donating ligands are central to this approach, which has been successfully applied to developing octahedral chiral-at-metal catalysts from metals like iridium, rhodium, ruthenium, osmium, and even iron. Recently, Shionoya extended this concept by designing a tetrahedral chiral-at-zinc catalyst.

By eliminating the need for chiral motifs in the ligand sphere, untapped possibilities arise for designing chiral metal catalysts with unconventional ligand environments, opening the door to catalysts with novel properties. This has been demonstrated by us and others through a multitude of applications. In our opinion this is particularly well illustrated by bis-cyclometalated iridium(iii) and rhodium(iii) catalysts, which uniquely combine photochemical activation with asymmetric Lewis acid catalysis. The helical chirality of these catalysts, along with the configurational inertness of the iridium and rhodium centers, makes the use of chiral ligands unnecessary and even counterproductive.

A critical factor in designing such chiral-at-metal catalysts is the configurational stability of the metal stereocenter. This explains why most up to date reported chiral-at-metal catalysts are octahedral 18-electron complexes from noble metals with a low-spin d^6^ electron configuration, offering optimal ligand field stabilization and high configurational stability. The main challenge for these d^6^-metal complexes is balancing inert ligands, which stabilize the metal configuration, with labile ligands, which provide reactivity for substrate or reagent coordination. In many systems, this balance can be achieved by including very strong σ-donor ligands. These σ-donor ligands are typically part of a multidentate structure and labilize monodentate ligands in the *trans*-position *via* the *trans*-effect, while simultaneously increasing the ligand field, thereby enhancing the inertness of the multidentate ligands. This straightforward and general approach can even be extended to more labile 3d-metal complexes, such as chiral-at-iron catalysts.

Future research on chiral-at-metal catalysis will likely see more economic methods for synthesizing chiral-at-metal complexes,^165^ an extension to other metals including earth abundant and non-precious metals,^166^ the introdution of new ligand frameworks, the discovery of new catalytic transformations, and the application to the synthesis of pharmaceuticals.

We hope this review inspires new approaches for designing chiral-at-metal catalysts by exploring various other metals, ligand classes, and alternative coordination topologies.

## Data availability

No primary research results, software or code have been included and no new data were generated or analyzed as part of this review.

## Conflicts of interest

The authors declare no conflict of interest.
